# Exenatide once weekly for alcohol use disorder investigated in a randomized, placebo-controlled clinical trial

**DOI:** 10.1172/jci.insight.159863

**Published:** 2022-10-10

**Authors:** Mette Kruse Klausen, Mathias Ebbesen Jensen, Marco Møller, Nina Le Dous, Anne-Marie Østergaard Jensen, Victoria Alberte Zeeman, Claas-Frederik Johannsen, Alycia Lee, Gerda Krog Thomsen, Julian Macoveanu, Patrick MacDonald Fisher, Matthew Paul Gillum, Niklas Rye Jørgensen, Marianne Lerbæk Bergmann, Henrik Enghusen Poulsen, Ulrik Becker, Jens Juul Holst, Helene Benveniste, Nora D. Volkow, Sabine Vollstädt-Klein, Kamilla Woznica Miskowiak, Claus Thorn Ekstrøm, Gitte Moos Knudsen, Tina Vilsbøll, Anders Fink-Jensen

**Affiliations:** 1 Psychiatric Centre Copenhagen, Rigshospitalet, Copenhagen, Denmark.; 2 Department of Clinical Medicine, Faculty of Health and Medical Sciences, University of Copenhagen, Copenhagen, Denmark.; 3 Department of Addictive Behavior and Addiction Medicine, Central Institute of Mental Health, and; 4 Mannheim Center for Translational Neurosciences, Medical Faculty of Mannheim, University of Heidelberg, Mannheim, Germany.; 5 Neurobiology Research Unit, Copenhagen University Hospital Rigshospitalet, Copenhagen, Denmark.; 6 Novo Nordisk Foundation Center for Basic Metabolic Research and Department of Biomedical Sciences and; 7 Department of Clinical Biochemistry, Centre of Diagnostic Investigation, University of Copenhagen, Copenhagen, Denmark.; 8 Department of Biochemistry and Immunology, University Hospital of Southern Denmark, Vejle, Denmark.; 9 Department of Clinical Pharmacology, Bispebjerg/Frederiksberg Hospital, University Hospital Copenhagen, Copenhagen, Denmark.; 10 National Institute of Public Health, University of Southern Denmark and University of Copenhagen, Copenhagen, Denmark.; 11 Department of Anesthesiology and Pediatric Anesthesiology, Yale University, New Haven, Connecticut, USA.; 12 National Institute on Alcohol Abuse and Alcoholism, National Institutes of Health, Bethesda, Maryland, USA.; 13 Department of Psychology,; 14 Department of Public Health, Section of Biostatistics, and; 15 Steno Diabetes Center Copenhagen, University of Copenhagen, Copenhagen, Denmark.

**Keywords:** Clinical Trials, Neuroscience, Addiction, Neuroimaging, Pharmacology

## Abstract

**Background:**

Alcohol use disorder (AUD) is a chronic, relapsing brain disorder that accounts for 5% of deaths annually, and there is an urgent need to develop new targets for therapeutic intervention. The glucagon-like peptide-1 (GLP-1) receptor agonist exenatide reduces alcohol consumption in rodents and nonhuman primates, but its efficacy in patients with AUD is unknown.

**Methods:**

In a randomized, double-blinded, placebo-controlled clinical trial, treatment-seeking AUD patients were assigned to receive exenatide (2 mg subcutaneously) or placebo once weekly for 26 weeks, in addition to standard cognitive-behavioral therapy. The primary outcome was reduction in number of heavy drinking days. A subgroup also completed functional MRI (fMRI) and single-photon emission CT (SPECT) brain scans.

**Results:**

A total of 127 patients were enrolled. Our data revealed that although exenatide did not significantly reduce the number of heavy drinking days compared with placebo, it significantly attenuated fMRI alcohol cue reactivity in the ventral striatum and septal area, which are crucial brain areas for drug reward and addiction. In addition, dopamine transporter availability was lower in the exenatide group compared with the placebo group. Exploratory analyses revealed that exenatide significantly reduced heavy drinking days and total alcohol intake in a subgroup of obese patients (BMI > 30 kg/m^2^). Adverse events were mainly gastrointestinal.

**Conclusion:**

This randomized controlled trial on the effects of a GLP-1 receptor agonist in AUD patients provides new important knowledge on the effects of GLP-1 receptor agonists as a novel treatment target in addiction.

**Trial registration:**

EudraCT: 2016-003343-11. ClinicalTrials.gov (NCT03232112).

**Funding:**

Novavi Foundation; Research Foundation, Mental Health Services, Capital Region of Denmark; Research Foundation, Capital Region of Denmark; Ivan Nielsen Foundation; A.P. Moeller Foundation; Augustinus Foundation; Woerzner Foundation; Grosserer L.F. Foghts Foundation; Hartmann Foundation; Aase and Ejnar Danielsen Foundation; P.A. Messerschmidt and Wife Foundation; and Lundbeck Foundation.

## Graphical abstract

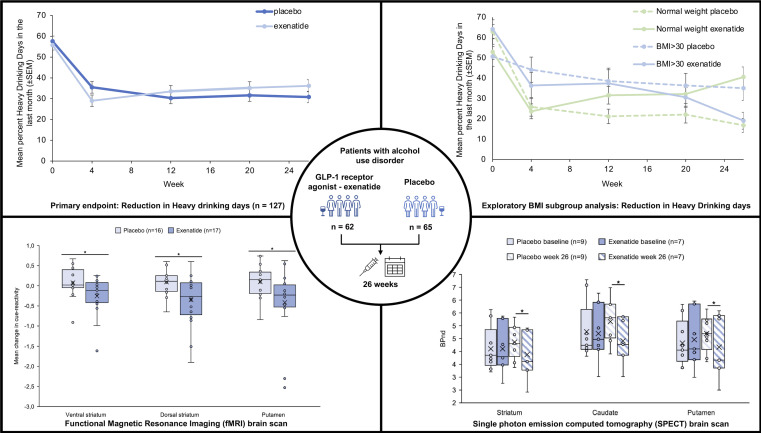

## Introduction

Alcohol use disorder (AUD) is an essential contributor to the burden of global disease (1). In Denmark, the cumulative all-cause 10-year mortality risk is almost 30% after a first-time hospital contact due to an alcohol problem (2). Only 3 medications are approved by the US Food and Drug Administration (FDA) to treat AUD: disulfiram, naltrexone, and acamprosate (3). About 40% of patients treated for AUD relapse within the first 3 years (4), and new targets for therapeutic interventions are urgently needed for this devastating chronic disease (1, 3).

The endogenous glucagon-like peptide-1 (GLP-1) is a 30–amino acid peptide hormone produced in the intestinal L cells in response to food intake (5), as well as in the nucleus tractus solitarius of the medulla oblongata (6). GLP-1 stimulates insulin secretion, inhibits glucagon secretion, and, notably, dampens appetite and food intake (5). GLP-1 receptor agonists are approved by the European Medicines Agency (EMA) and FDA to treat type 2 diabetes and obesity (7). Since drugs of abuse and alcohol activate the same reward system that underlies food reward (8), it is conceivable that appetite-regulating peptides such as GLP-1 target areas associated with reward and addiction. In support of this hypothesis, several studies have reported expression of GLP-1 receptors in brain areas associated with reward and addiction (6, 9–16). Furthermore, treatment with GLP-1 receptor agonists reduce alcohol intake and decrease relapse-like alcohol drinking in nonhuman primates (17) and rodents (18). In humans, a recent study reported that the GLP-1 receptor 168Ser allele variant was associated with increased alcohol intake in humans (19). However, the effects of a GLP-1 receptor agonist on alcohol consumption in humans remain unknown. To this end, we performed a randomized, placebo-controlled clinical trial lasting 26 weeks plus a long-term 6-month follow-up to evaluate the efficacy of the once-weekly GLP-1 receptor agonist exenatide (Bydureon) at a dose of 2 mg in patients diagnosed with AUD according to the Diagnostic and Statistical Manual of Mental Disorders (DSM-5). In total, 127 treatment-seeking AUD patients, who had a minimum of 5 heavy drinking days, i.e., 60/48 g of alcohol or more per day (men/women) in the past 30 days, were included. Since the pharmacodynamics and pharmacokinetics of a GLP-1 receptor agonist in patients with AUD have not been investigated, we chose a dosing regimen consistent with established tolerability and efficacy in treatment of type 2 diabetes, i.e., exenatide, 2 mg subcutaneously once weekly. Importantly, exenatide crosses the blood-brain barrier (20), and a similar dosing regimen, i.e., 2 mg subcutaneously once weekly, has recently shown efficacy in other neuropsychiatric disorders, including nicotine dependence (21) and Parkinson’s disease (20), suggesting a central engagement, possibly mediated, at least in part, by dopamine signaling (22).

The primary endpoint was reduction in heavy drinking days, recorded with the Time-Line Follow Back method (23). A subgroup of the patients had a functional MRI (fMRI) scan and a single-photon emission CT (SPECT) scan performed at baseline and at week 26. Using the fMRI technique, we investigated whether exenatide once weekly would reduce alcohol cue reactivity in brain areas involved in drug reward and addiction, and in top-down regulation of impulsivity (24), as preclinical and clinical evidence suggests that GLP-1 receptor stimulation may be associated with improved cognitive impairment (25). By use of the SPECT scan, we measured the availability of the striatal dopamine transporter (DAT), a key modulator of extracellular dopamine. Dopamine plays a pivotal role in the neurobiological underpinnings of reward (26), and a large body of evidence suggests that brain dopamine homeostasis changes following chronic alcohol intake (27).

## Results

### Characteristics of the patients.

**Figure 1:**
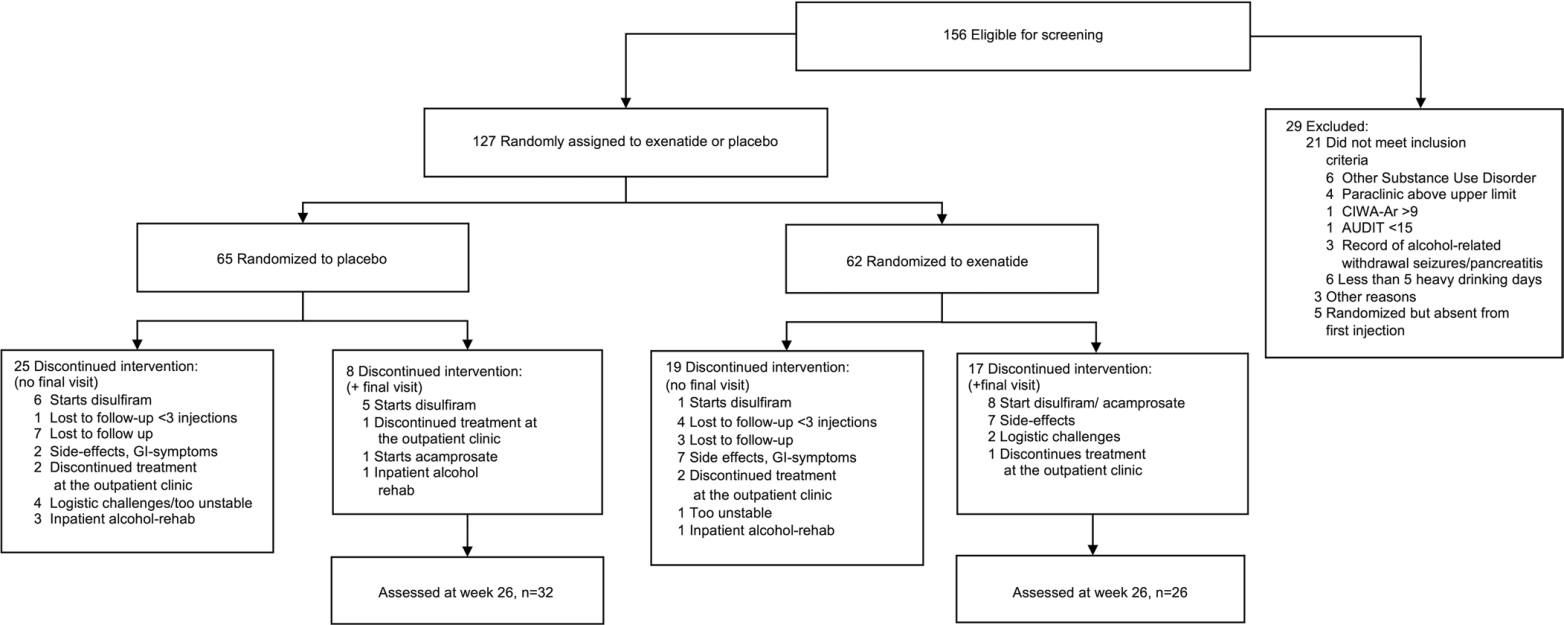
CONSORT flow diagram. Study diagram of patient flow according to CONSORT 2010 statement. Details regarding initial meetings and ineligibility for screening can be found in Supplemental Figure 3, and a flowchart for the 6-month follow-up can be found in Supplemental Figure 1. Of the 127 patients included in the study, 65 patients were randomized to 2 mg exenatide once weekly, and 62 patients were randomized to placebo. Thirty-two patients from the exenatide group and 26 patients from the placebo group completed the study after 26 weeks of trial participation. AUDIT, Alcohol Use Disorders Identification Test; CIWA-Ar, Clinical Institute Withdrawal Assessment for Alcohol, Revised.

**Figure 2:**
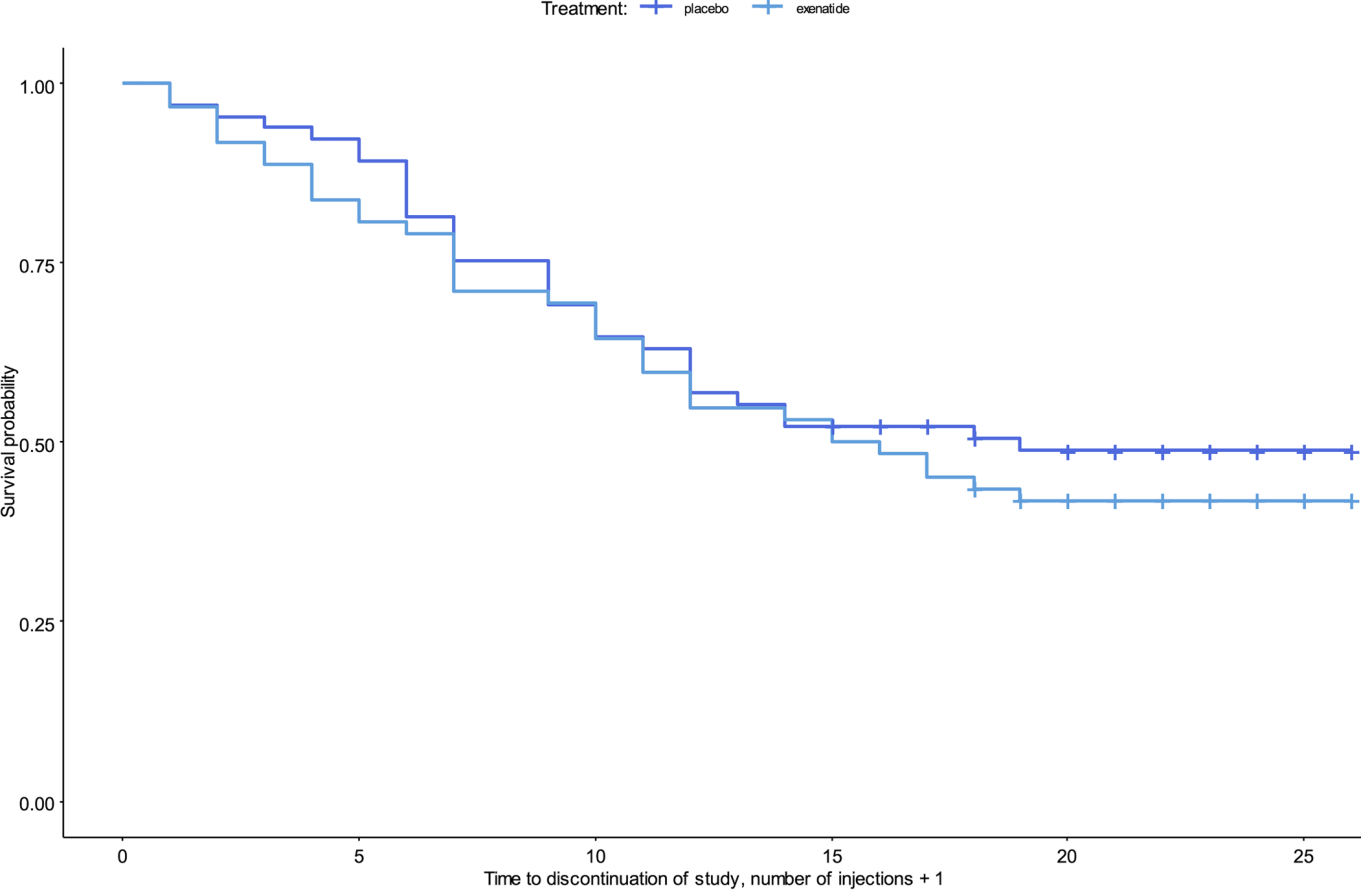
Kaplan-Meier survival curve of patients who withdrew from the trial or were lost to follow-up. The time to discontinuation was not significantly different in the 2 groups (*P* = 0.46). Input data are the number of injections + 1 because patients were registered as discontinued in the week after the last injection was received. All patients are included (*n* = 127). Censoring is indicated by the + mark.

From August 7, 2017, to October 1, 2019, 152 patients were screened for eligibility, and 127 patients were enrolled; 62 were randomly assigned to the exenatide group, and 65 were assigned to the placebo group (Figure 1). Overall, the 2 treatment groups were balanced with respect to baseline characteristics (Table 1). All patients were White, with a mean age of 52 years. The majority of the patients were men (60%). On average, they had 17 heavy drinking days and an overall alcohol intake of 2,400 g of pure alcohol over the last month, and 80% fulfilled the criteria for severe AUD, i.e., more than 5 symptoms, according to DSM-5 (see baseline characteristics and flowchart for the patients included in the brain imaging substudy in Supplemental Appendices 1 and 2; supplemental material available online with this article; https://doi.org/10.1172/jci.insight.159863DS1). Of the 127 patients included, a total of 58 patients completed the trial, i.e., participated in the last follow-up after 26 weeks of treatment; 25 patients finished prematurely, i.e., participated in a final examination before 26 weeks of treatment. Fifty-five patients participated in the long-term 6-month follow-up visit (Supplemental Figure 1), with the last visit held on the October 10, 2020. The mean (SD) number of injections was 22.6 (2.2) in the exenatide group and 22.1 (2.8) in the placebo group (Supplemental Table 1). There was no difference (*P* = 0.46) between the 2 groups in time to trial discontinuation (Figure 2). In addition, 25 healthy controls matched for sex, age, and educational status of the included patients were recruited for the fMRI substudy.

### Efficacy.

**Figure 3:**
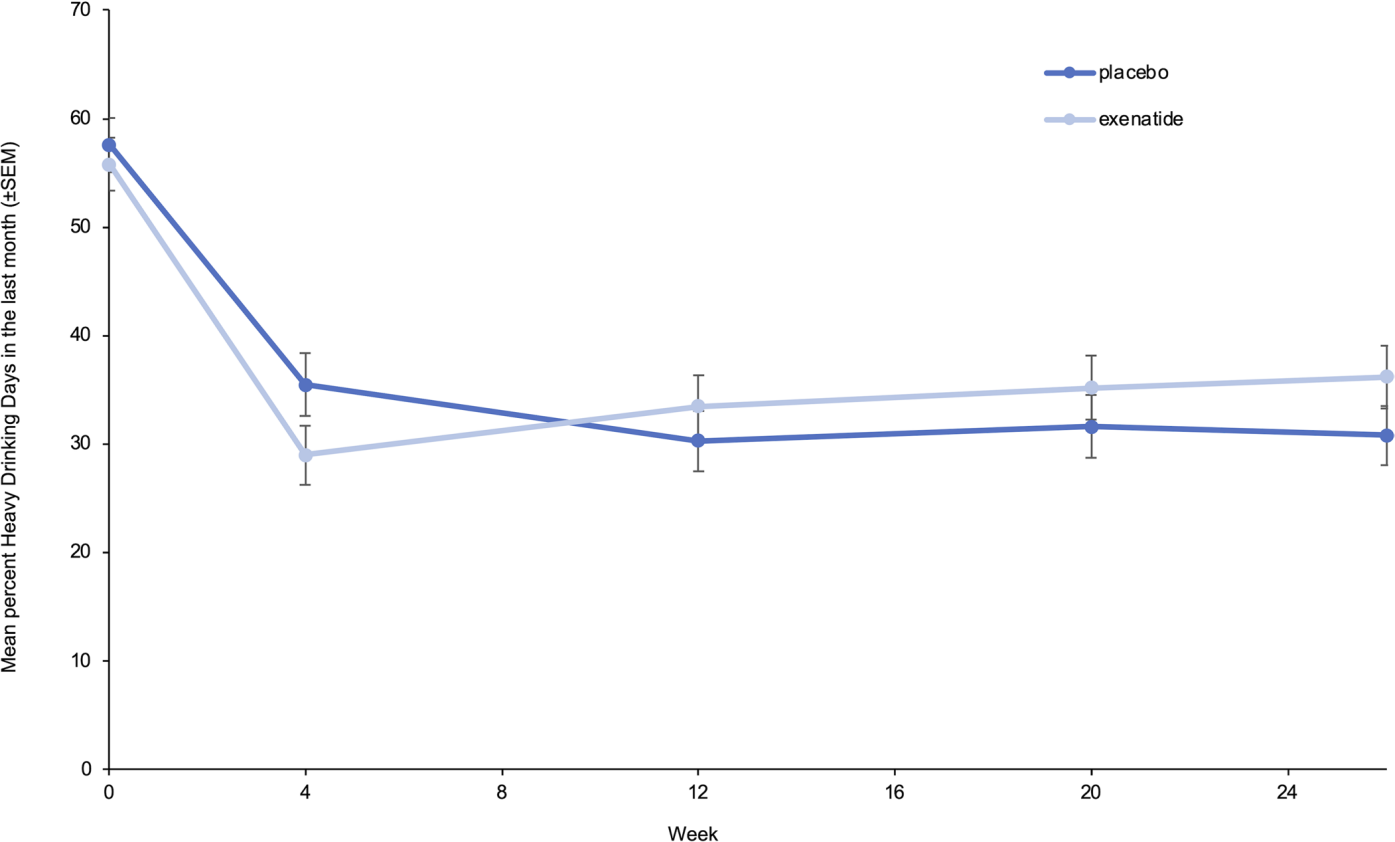
Reduction in heavy drinking days. Mean percentage heavy drinking days in the last 30 days, measured with the Time-Line Follow Back (TLFB) method, at all assessments (week 0, week 4, week 12, week 20, week 26). Data were analyzed with an ANOVA adjusted for baseline, and missing data were imputed with the use of multiple imputations as described in the text (*n* = 127). Data represent mean ± SEM.

For both groups, the number of heavy drinking days (Table 2 and Figure 3) and total alcohol intake (Table 2) were strongly reduced, but there were no significant differences between the 2 groups. The exenatide group had a reduction in BMI of 0.95 (95% CI, –1.6 to –0.3, *P* = 0.006), glycated hemoglobin (HbA1c) of 1.6 mmol/mol (95% CI, –2.8 to –0.4, *P* = 0.011), and a worsening in Drug Use Disorders Identification Test (DUDIT) score of 0.96 points (95% CI, 0.7 to 1.3, *P <* 0.001) relative to the placebo group (Table 2). There were no group differences in FGF-21, phosphatidylethanol, or bone markers (Table 2); life quality measurements, i.e., 36-Item Short Form Health Survey (SF-36) (Supplemental Table 3) and Symptom Checklist-92 (SCL-92) (Supplemental Table 4); or cognition i.e., Screen for Cognitive Impairment in Psychiatry test (SCIP) (Supplemental Table 5). Exenatide once weekly increased urine oxidative stress parameters — 8-oxoGuo of 0.24 nmol/mmol creatinine (95% CI, 0.04 to 0.44, *P* = 0.022) and 8-oxodG of 0.43 nmol/mmol creatinine (95% CI, 0.15 to 0.72, *P* = 0.003) — relative to placebo (Supplemental Table 2). In the exenatide group, the plasma level of exenatide was 45.6 pmol/L (95% CI, 16.5 to 74.7, *P* = 0.003), and the overall anti-exenatide antibody binding was 16.1% (95% CI, 6.9 to 25.3, *P* = 0.002) relative to the placebo group (Supplemental Table 2).

### Exploratory analyses.

**Figure 4:**
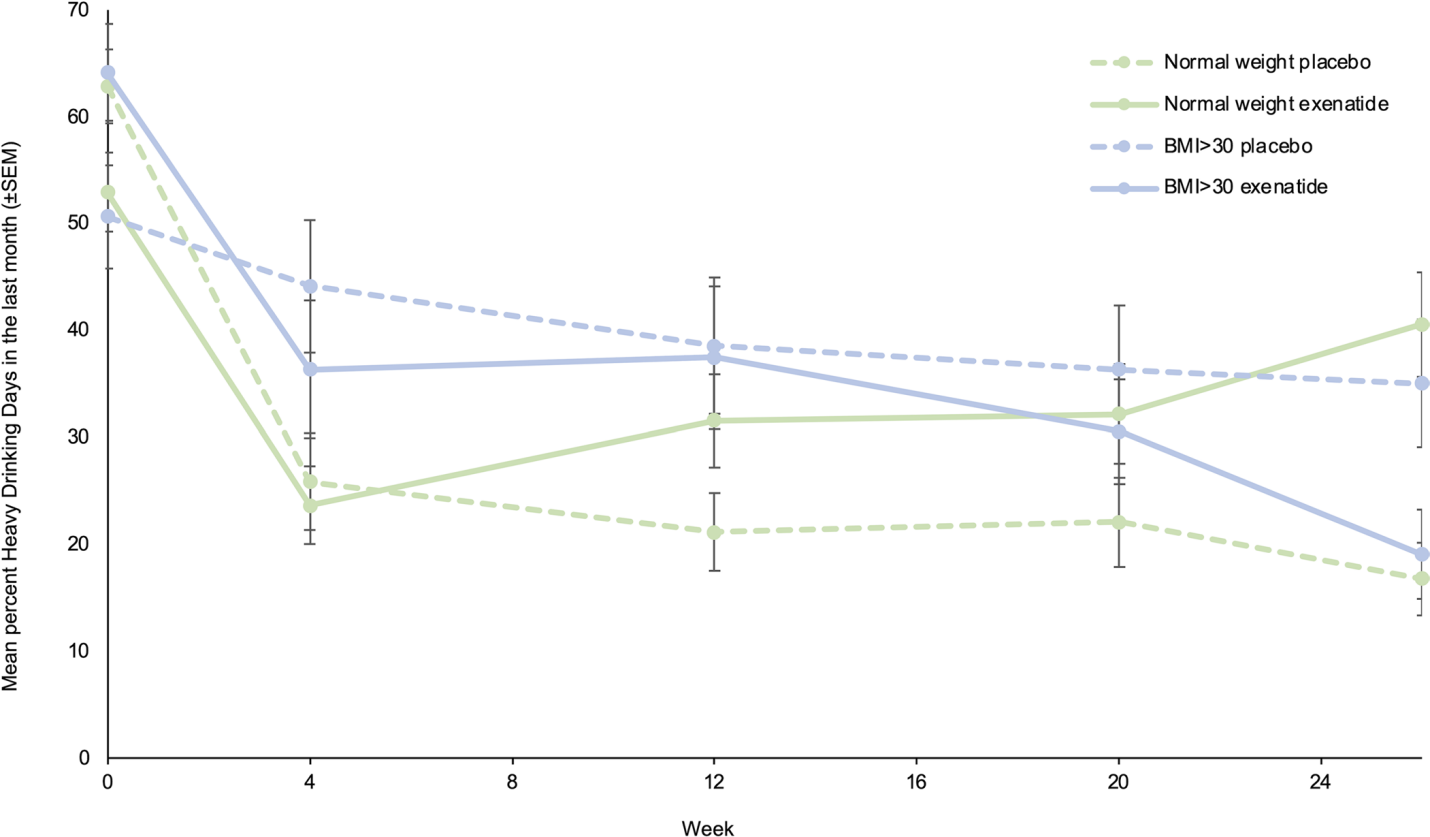
Reduction in heavy drinking days in BMI subgroups. Exploratory analysis of mean percentage heavy drinking days in the last 30 days, measured with the TLFB method, at all assessments (week 0, week 4, week 12, week 20, week 26) within the BMI subgroups. Normal weight, *n* = 52; BMI > 30, *n* = 30. Only significant findings from Supplemental Table 7 are included. Data were analyzed with an ANOVA adjusted for baseline, and missing data were imputed with the use of multiple imputations as described in the text. Data represent mean ± SEM.

**Figure 5:**
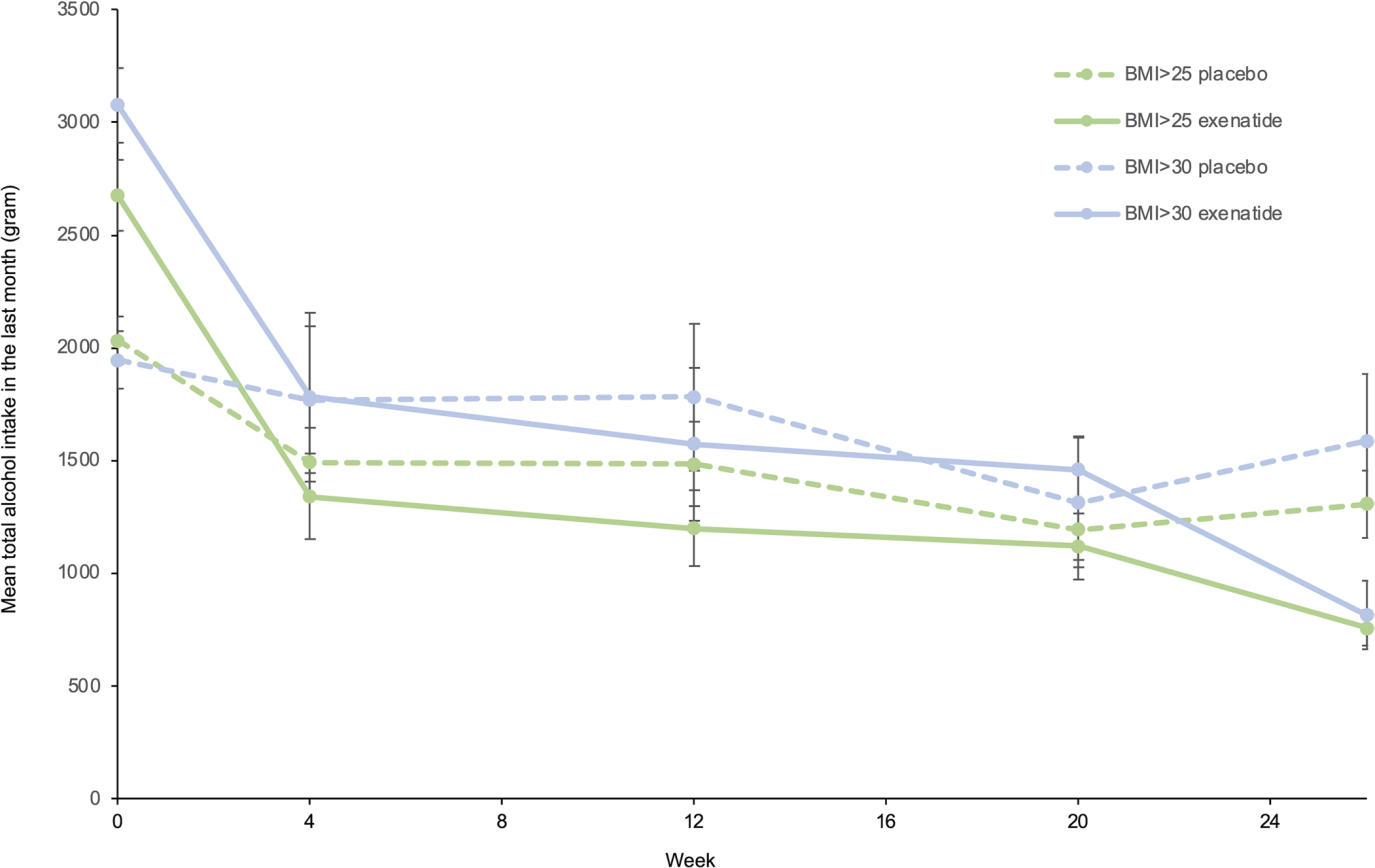
Reduction in total alcohol intake in BMI subgroups. Exploratory analysis of mean total alcohol intake in the last 30 days, measured with the TLFB method, at all assessments (week 0, week 4, week 12, week 20, week 26) within the BMI subgroups. BMI > 25, *n* = 75; BMI > 30, *n* = 30. Only significant findings from Supplemental Table 7 are included. Data were analyzed with an ANOVA adjusted for baseline, and missing data were imputed with the use of multiple imputations as described in the text. Data represent mean ± SEM.

Exenatide once weekly did not reduce the number of heavy drinking days in the prespecified subgroup analyses (baseline heavy drinking days, severity of DSM-5 criteria, and geography) (Supplemental Table 6). However, an exploratory subgroup analysis (Supplemental Table 7) including BMI subgroups revealed that in obese patients with a BMI greater than 30 kg/m^2^ (*n* = 30), exenatide reduced heavy drinking days by 23.6 percentage points (95% CI, –44.4 to –2.7, *P* = 0.034) (Figure 4) and reduced total alcohol intake per 30 days by 1,205 g (95% CI, –2,206 to –204, *P* = 0.026) relative to placebo (Figure 5). In patients with a BMI less than 25 kg/m^2^ (*n* = 52), treatment with exenatide increased number of heavy drinking days by 27.5 percentage points (95% CI, 4.7 to 50.2, *P* = 0.024) relative to the placebo group. However, in this subgroup (BMI < 25 kg/m^2^) the total alcohol intake did not differ between treatment groups. Other exploratory post hoc subgroup analyses were performed to investigate whether some subgroups responded differently from others on the intervention. However, no significant differences were observed with respect to sex, baseline craving (Penn Alcohol Craving Scale score), baseline Alcohol Use Disorders Identification Test (AUDIT) score, baseline number of days without alcohol, baseline total alcohol consumption, fMRI subgroup (*n* = 22), and SPECT subgroup (*n* = 16).

Besides the exploratory subgroup analyses, we also looked at the reduction in WHO risk drinking levels (28). Both groups reduced their risk drinking levels, but there was no significant difference between the 2 groups (Supplemental Table 6).

To explore whether there was a correlation between change in HbA1c and change in heavy drinking days, the Pearson correlation coefficient was computed in the imputed data set (*n* = 127) to assess linear relationship. Here, we found a weak negative correlation between the 2 variables [*r*(12755) = –0.27, *P* = 0.001]. We also found a weak negative correlation between changes in HbA1c and changes in total alcohol intake [*r*(12755) = –0.36, *P* = 0.001].

### Six-month long-term follow-up.

There was no difference between the 2 groups at the 6-month follow-up after exenatide or placebo discontinuation (Table 2 and Supplemental Table 8), except for a higher AUDIT score (5.1 points; 95% CI, 0.9 to 9.3, *P* = 0.02) in the original exenatide group (adjusted from the end of treatment) compared with the placebo group.

### fMRI alcohol cue reactivity.

**Figure 6:**
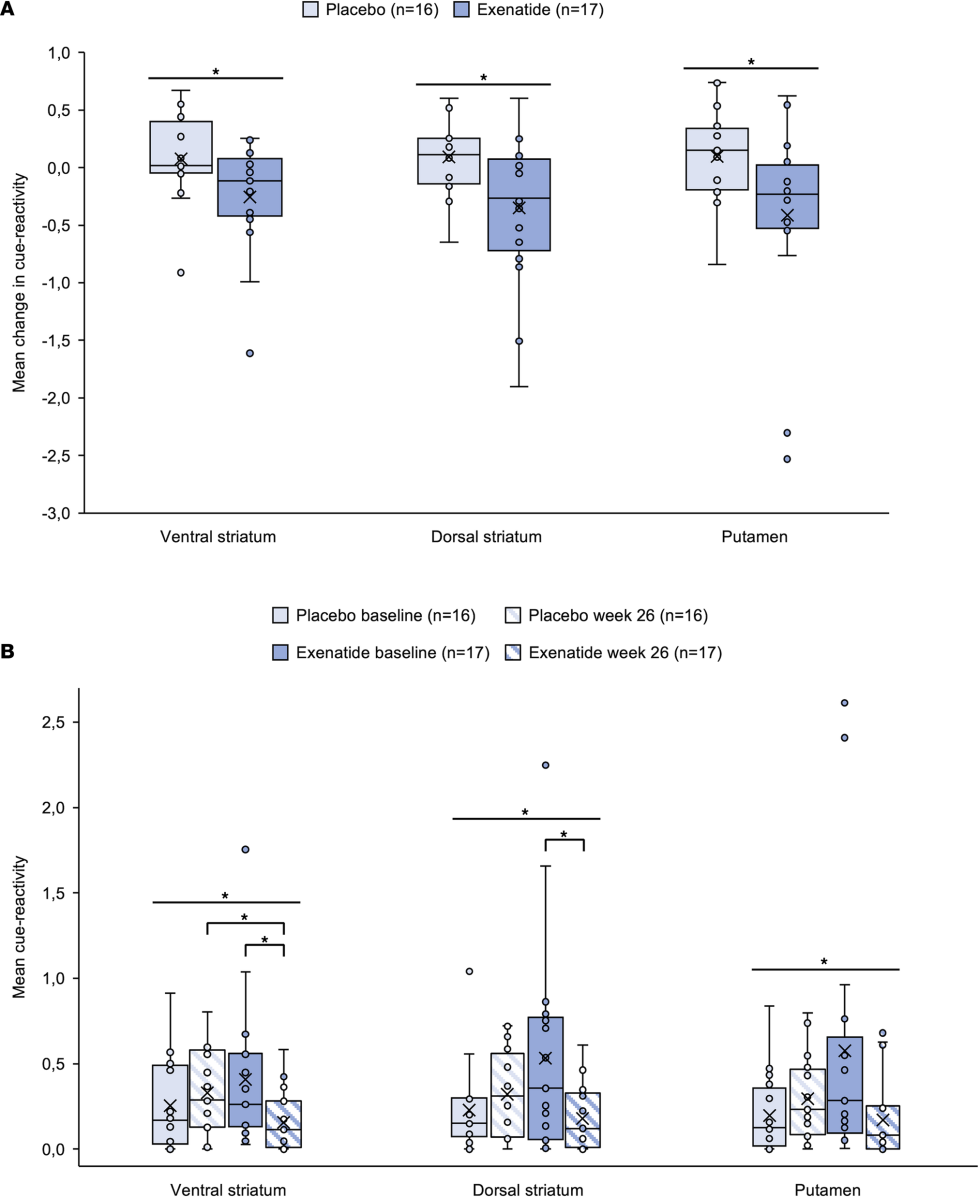
fMRI ALCUE ROI results. (**A**) Key fMRI findings showed reduced cue reactivity after 26 weeks of treatment with exenatide compared with placebo. Analysis revealed statistically significant interaction between the treatment and time on fMRI response in all 3 ROIs: ventral striatum [*F*(1,31) = 4.744, *P* = 0.037, partial η^2^ =0.133], dorsal striatum [*F*(1,31) = 6.124, *P* = 0.019, partial η^2^ = 0.165], putamen [*F*(1,31) = 4.730, *P* = 0.037, partial η^2^ = 0.132]. *P < 0.05. (**B**) In more detail, we found that at week 26, cue-induced activity was significantly lower in ventral striatum after treatment with exenatide compared with placebo (*M* = –0.176, SE = 0.075, *P* = 0.025), but not in dorsal striatum (*M* = –0.142, SE = 0.076, *P* = 0.073) nor in putamen (*M* = –0.123, SE = 0.084, *P* = 0.153). At baseline, cue-induced activity did not differ significantly between groups. Within the exenatide group, cue-induced activity was significantly reduced from baseline to week 26 in ventral striatum (*M* = –0.254, SE = 0.116, *P* = 0.044) and in dorsal striatum (*M* = –0.351, SE = 0.156, *P* = 0.039), but not in putamen (*M* = –0.405, SE = 0.202, *P* = 0.063). Within the placebo group, no statistically significant differences were found. (**A** and **B**) ROI data were analyzed using a repeated-measures ANOVA including factors group and time and an independent sample 2-tailed *t* test comparing groups (placebo and exenatide). Placebo, *n* = 16; exenatide, *n* = 17. Boxes represent upper and lower quartiles, the line represents the median, and the X represents the mean. Horizontal lines indicate significant interactions between treatment and time (*P < 0.05), and brackets indicate significant simple effects (*P < 0.05).

The predefined region of interest (ROI) masks were acquired from WFU PickAtlas (29). The analyses revealed a statistically significant interaction between treatment and time on the fMRI response in all 3 ROIs: ventral striatum [*F*(1,31) = 4.744, *P* = 0.037, partial η^2^ = 0.133], dorsal striatum [*F*(1,31) = 6.124, *P* = 0.019, partial η^2^ = 0.165], and putamen [*F*(1,31) = 4.730, *P* = 0.037, partial η^2^ = 0.132], indicating reduced cue reactivity after 26 weeks of treatment with exenatide compared with placebo. The ROI analysis in the caudate and nucleus accumbens did not reveal any significant effects (Figure 6A). At week 26, the cue-induced activity in ventral striatum was significantly lower in the exenatide group compared with placebo (mean difference [*M]* = –0.176, SEM = 0.075, *P* = 0.025). However, in the dorsal striatum (*M* = –0.142, SEM = 0.076, *P* = 0.073) and in the putamen (*M* = –0.123, SEM = 0.084, *P* = 0.153) no significant differences were observed. At baseline, cue-induced activity did not differ between the treatment groups. Within the exenatide group, cue-induced activity was significantly reduced from baseline to week 26 in ventral striatum (*M* = –0.254, SEM = 0.116, *P* = 0.044) and in dorsal striatum (*M* = –0.351, SEM = 0.156, *P* = 0.039), but not in putamen (*M* = –0.405, SEM = 0.202, *P* = 0.063). Within the placebo group, we found no statistically significant differences (Figure 6B).

**Figure 7:**
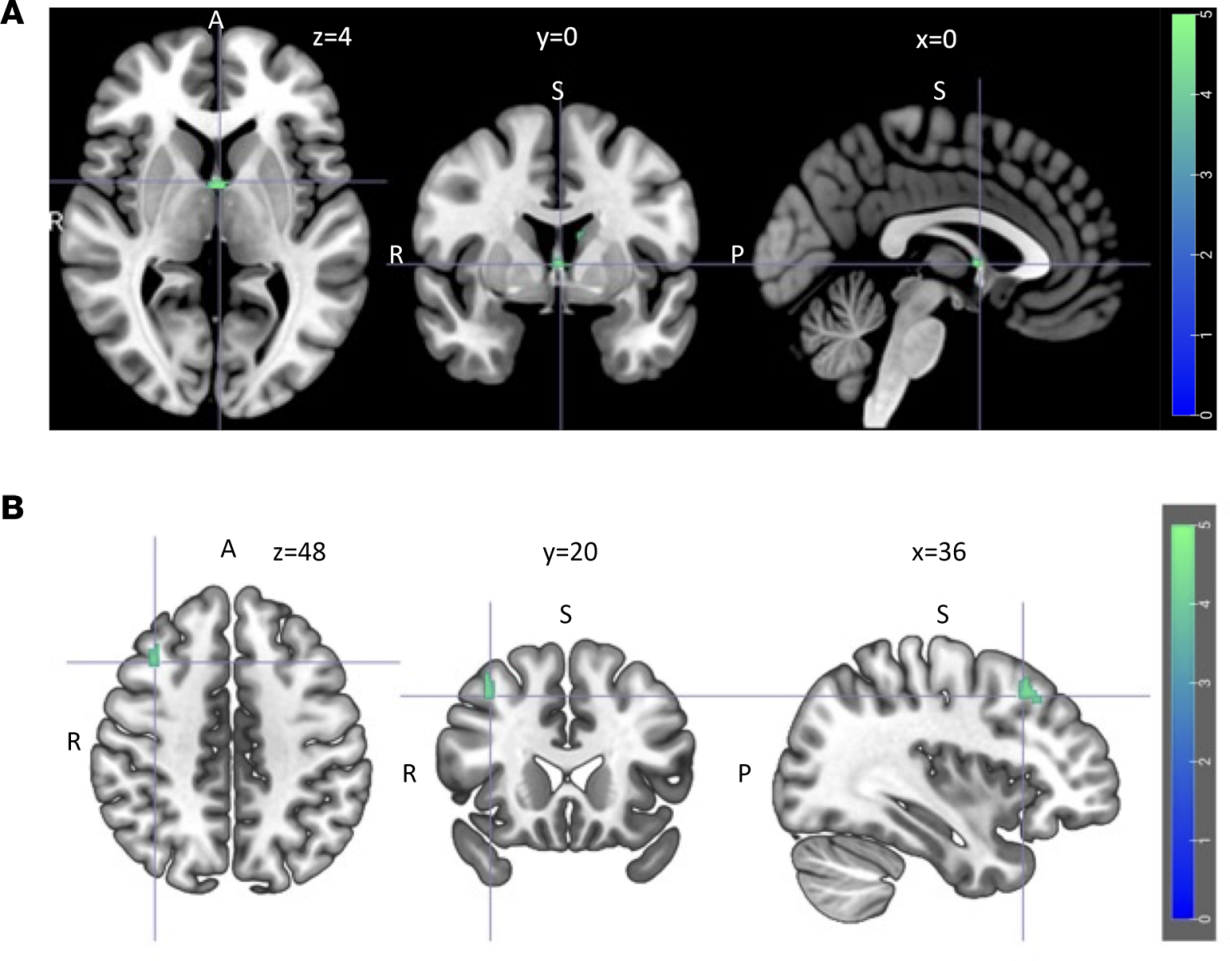
fMRI ALCUE whole-brain results. Reduced cue-induced activation in the exenatide group compared with the placebo group after 26 weeks of treatment in the left caudate and septal area (*x*, *y*, *z* coordinates = 0, 0, 4) (**A**) and right middle frontal gyrus (*x*, *y*, *z* coordinates = 36, 20, 48) (**B**). A 2-sample 2-tailed *t* test was performed for the post hoc analyses to compare groups (placebo, exenatide) and within a group across time (placebo/exenatide: T1, T2). For the group comparisons, the contrast of interest used was ‘alcohol > neutral stimuli’, where the probability of a family wise error (FWE) was set to 0.05 to control for multiple statistical testing. Using the AlphaSim (3dClustSim) method, a combined voxel wise threshold of *P* < 0.001 and a cluster extent threshold of 101 voxels were calculated (*n* = 22).

At baseline, the exploratory whole-brain analysis showed no significant difference in cue reactivity between the placebo group and the exenatide group. When cue reactivity in all patients was compared with that in healthy controls, significant differences were found in the left superior and middle frontal gyrus, caudate, and insula (*P* = 0.001). However, at the week 26 rescan, these differences were no longer significant. At the week 26 assessment, cue-induced activation was significantly reduced in the exenatide group compared with the placebo group in the following brain areas (Supplemental Appendix 1: Supplemental Table 13): left caudate and septal area (Figure 7A) and right middle frontal gyrus (Figure 7B). There was no significant change in cue reactivity in the placebo group at the rescan, but the exenatide group showed a significant reduction in cue-induced activation in the temporal lobe, hippocampus, and parahippocampus (rescans per protocol: Supplemental Appendix 1: Supplemental Table 14, Supplemental Figure 5; rescans per protocol including premature rescans: Supplemental Appendix 1: Supplemental Table 15, Supplemental Figure 6).

### Subjective craving results: fMRI Subjective craving results.

The analysis showed a significant difference at baseline between the healthy controls and the patients (*P <* 0.001; mean ± SD: healthy controls, 8.8 ± 15.96; placebo group, 33.5 ± 26.9; exenatide group, 30.6 ± 28.6). At the week 26 follow-up, this was no longer significant (*P* = 0.50; mean ± SD: healthy controls, 8.8 ± 15.96; placebo group, 13.6 ± 12.0; exenatide group, 14.8 ± 23.07), and there were no significant differences between the exenatide and the placebo group (*P* = 0.980).

### fMRI spatial working memory.

**Figure 8:**
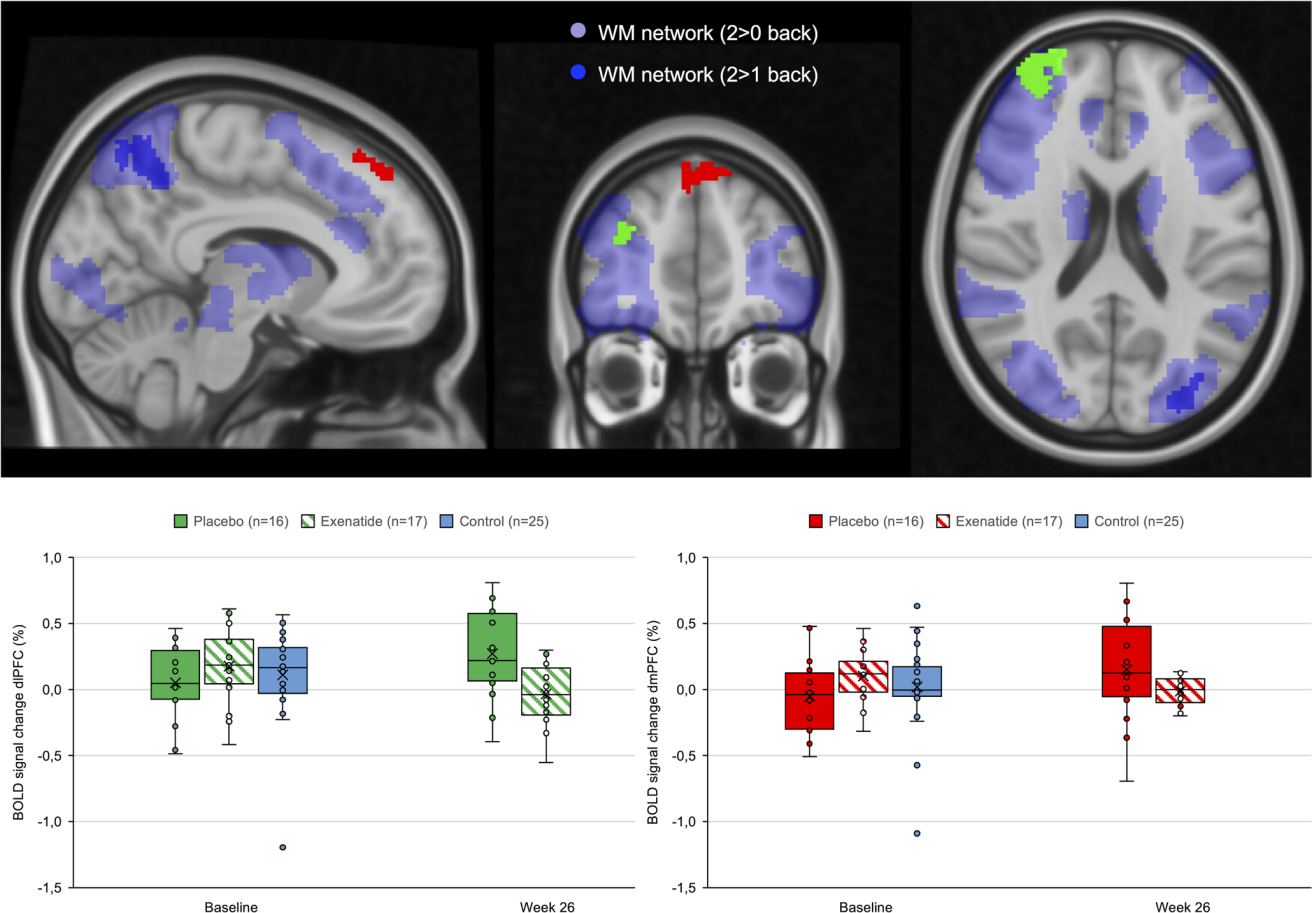
fMRI spatial working memory task (*N*-back task). The exenatide group showed a reduction at follow-up in the response to the 2-back > 1-back task compared with the placebo group (2-way mixed-effect ANOVA; placebo, *n* = 16; exenatide, *n* = 17; control, *n* = 25) in 2 prefrontal clusters (frontal pole *x*, *y*, *z* = 34, 54, 20, corrected *P <* 0.002; superior frontal gyrus *x*, *y*, *z* = 4, 46, 46, corrected *P <* 0.001). Boxes represent upper and lower quartiles, the line represents the median, and the X represents the mean. dlPFC, dorsolateral prefrontal cortex; dmPFC, dorsomedial prefrontal cortex.

The voxel-wise analysis showed a significant reduction in the exenatide group at the week 26 rescan compared with placebo in response to the 2-back > 1-back task in 2 clusters in the right frontal pole and right superior frontal gyrus, within the dorsolateral prefrontal cortex ROI (Figure 8 and Supplemental Appendix 1: Supplemental Table 16). The additional right dorsolateral prefrontal cortex ROI analysis showed no significant change in the exenatide group at week 26 compared with placebo in task-related activations [*F*(1,31), *P* = 0.122, partial η^2^ = 0.076]. The reduction in task-related neuronal activations in the exenatide group occurred in the absence of change in cognitive performance on the Screen for Cognitive Impairment in Psychiatry (*P* = 0.93).

### SPECT dopamine transporter availability.

**Figure 9:**
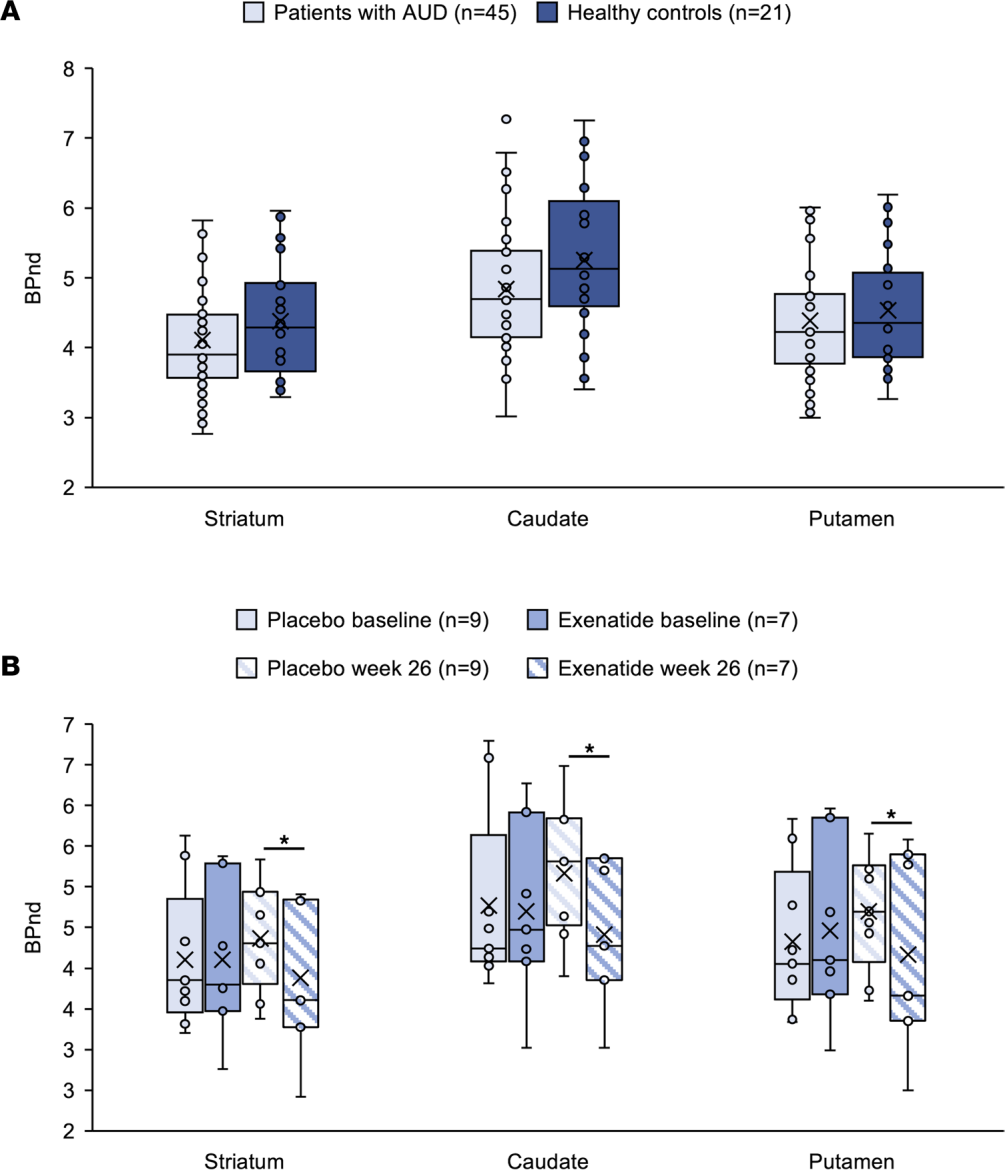
SPECT DAT results combined. (**A**) Baseline DAT availability in striatum, caudate, and putamen in AUD patients did not differ from that in healthy controls. Data were analyzed with a 1-way ANCOVA, adjusted for baseline DAT availability. Healthy controls, *n* = 21; patients at baseline, *n* = 45. (**B**) At the week 26 rescan, DAT availability in striatum, caudate, and putamen was significantly lower in the exenatide group compared with the placebo group [striatum, *F*(1,13) = 4.978, *P* = 0.044; caudate, *F*(1,13) = 8.066, *P* = 0.014; putamen, *F*(1,13) = 6.571, *P* = 0.024]. Data were analyzed with an ANCOVA adjusted for age. Placebo, *n* = 9; exenatide, *n* = 7. *P < 0.05. (**A** and **B**) Boxes represent upper and lower quartiles, the line represents the median, and the X represents the mean.

After adjustment for age, there were no significant differences comparing baseline DAT availability of the patients with AUD and healthy controls in striatum [*F*(1,62) = 0.474, *P* = 0.494], caudate [*F*(1,62) = 1.160, *P* = 0.286], and putamen [*F*(1,62) = 0.005, *P* = 0.944] (Figure 9A).

At the week 26 rescan, DAT availability in striatum, caudate, and putamen was significantly lower in the exenatide group compared with the placebo group [striatum: *F*(1,13) = 4.978, *P* = 0.044; caudate: *F*(1,13) = 8.066, *P* = 0.014; putamen: *F*(1,13) = 6.571, *P* = 0.024] (Figure 9B and Supplemental Appendix 2: Supplemental Table 18).

### Safety.

Gastrointestinal (GI) symptoms, body weight loss, fatigue, and injection site reactions were the most common adverse events reported, and the incidence was higher in the exenatide compared with the placebo group (nausea, 37.1% vs. 15.4%; decreased appetite, 24.2% vs. 9.2%; vomiting, 22.6% vs. 7.7%; overall weight loss, 67.7% vs. 40.0%; fatigue, 12.9% vs. 4.6%; injection site reaction, 41.0% vs. 0.0%) (Table 3). The GI side effects reported lasted until the first 5 weeks of treatment, and the weight loss continued throughout the trial. The injection site reactions were typically small nodules of 1–2 cm, hard, mobile, skin-colored, and were reabsorbed within 6 weeks, leaving no scar. Serious adverse events were reported almost equally between the 2 groups (exenatide 24.2% vs. placebo 18.5%), and there were no cases of acute pancreatitis or elevation of pancreas enzymes above upper limits. One patient in the exenatide treatment group committed suicide 7 weeks after withdrawal from the trial. One patient in the placebo group was found dead after being hospitalized 3 times in one week for alcohol withdrawal symptoms.

## Discussion

To our knowledge, this is the first randomized controlled trial (RCT) investigating the effects of exenatide in AUD patients. Treatment with exenatide once weekly was not superior to placebo in reducing the number of heavy drinking days in the prespecified analysis. The negative results could reflect the characteristics of the AUD patients included in our RCT. Data from preclinical trials showed that high-alcohol-consuming animals decreased their alcohol intake significantly more than low-alcohol-consuming animals when treated with a GLP-1 receptor agonist (23, 31). In the present trial, 80% of the patients fulfilled the DSM-5 criteria for severe AUD. However, their severity profile, based on baseline alcohol intake and heavy drinking days (Table 1), was less severe than those observed in other AUD pharmacotherapy trials (32, 33). Another explanation could be that the potent placebo response could have masked a possible beneficial effect of exenatide (Figures 3 and 4). The observed potent placebo response could be due to the standardized cognitive-behavioral therapy against AUD (34) offered to all participants in the study, but it could also be due to the lesser severity profile of the AUD patients included, which is typically linked to a higher placebo response (35). Large placebo responses are also reported in other clinical AUD trials and shown to be negatively correlated with the treatment intervention effect sizes (36).

Predefined fMRI brain ROI analysis found a reduced alcohol cue reactivity in the exenatide group compared with the placebo group in the ventral striatum, a region that plays a pivotal role in addiction and relapse (Figure 6). This finding is important because it implies that AUD subjects treated with exenatide lose the incentive salience of alcohol-associated cues. The exenatide-induced reduction in cue reactivity in the septal area (37) observed in the whole-brain analysis (Figure 7A) is particularly intriguing as this is an area connected to reward (15), and a brain area where GLP-1 receptors are highly expressed (6). These findings are in accordance with a central effect of exenatide as mentioned in the Introduction. Future fMRI studies investigating the effects of GLP-1 receptor agonists on alcohol cue–induced activation should include the septal area as a region of interest.

Impairments in cognitive processes related to executive function in AUD patients (38) may negatively influence clinical outcomes owing to deficits in self-regulation (39). In the fMRI spatial working memory test, we found reduced cue reactivity in the dorsal prefrontal cortex in the exenatide group compared with the placebo group, possibly indicating a reduced effort to maintain cognitive performance (40).

The SPECT substudy revealed no significant differences in DAT availability at baseline between the AUD patients and healthy controls, which is in accordance with some earlier findings (41), but in discordance with others (42). After 26 weeks of treatment, the analysis revealed a significant reduction of DAT in the striatum, caudate, and putamen in the exenatide group compared with placebo, which might compensate for the decreased dopamine activity previously reported in AUD patients (43). Notably, this effect is most likely not acutely induced, since no change in DAT availability was observed after acute treatment with exenatide in healthy volunteers (44).

Even though the results from the exploratory post hoc BMI subgroup analysis are preliminary, we think they are of substantial interest because overlapping dysfunctional brain circuits are observed in individuals with obesity or addiction (8), and deranged GLP-1 signaling is also reported in obese individuals (45). In addition, an fMRI study in obese versus lean individuals showed that exenatide infusions “normalized” the brain response to a food paradigm in obese patients with a BMI greater than 30 kg/m^2^ compared with lean individuals (46). Moreover, several GLP-1 receptor agonists have recently been approved to treat obesity (BMI > 30 kg/m^2^), and other compounds are under development (7). The reason why the number of heavy drinking days was increased in the subgroup of exenatide-treated patients with a BMI less than 25 kg/m^2^ compared with placebo-treated patients could be that those lean individuals treated with exenatide experienced a larger decrease in blood sugar (47), and this might be associated with increased alcohol craving (48).

The significant increase in urinary oxidative stress markers in the exenatide group was previously reported in type 2 diabetes patients treated with exenatide (49), but the clinical significance of rising levels of urinary stress parameters 8-oxoGuo and 8-oxodG is currently unknown (50). Notably, increased urinary oxidative stress parameters in patients with type 2 diabetes are associated with increased mortality risk (51), and the clinical impact of these biomarkers should be further investigated.

GLP-1 receptor agonists have shown beneficial skeletal effects in rodents (52). However, in the present trial, no differences in bone turnover markers were observed between groups, indicating that bone-related adverse effects are not of concern in this patient population.

Both the exenatide group and the placebo group exhibited an overall reduction in DUDIT score after 26 weeks of treatment. However, the exenatide group had a significantly higher DUDIT score compared with placebo after 26 weeks of treatment (Table 2). An exclusion criterion was a diagnosis of any active substance use disorder (SUD) except for nicotine. Men with a DUDIT score greater than 6 and women with a DUDIT score greater than 2 were screened according to International Classification of Diseases, Tenth Revision (ICD-10), SUD criteria and, if diagnosed with SUD, excluded from the trial. Only 4 of the 25 included participants with a positive DUDIT score (range between 1 and 22 points) finished per protocol. This is essential information for a follow-up study, where it may be relevant to exclude all individuals with a positive baseline DUDIT score to increase study compliance.

The previously reported safety profile of exenatide once weekly is consistent with the present safety data. Our most significant safety concern was the risk of pancreatitis in patients with AUD (3) combined with the associated risk of exenatide treatment (53, 54). Importantly, none of the patients experienced a rise in blood amylase above upper limits or developed pancreatitis. Surprisingly, the injection site reactions to exenatide were a bigger problem for the patients, due to unexpected concerns from their relatives, who might have been unaware of their AUD diagnosis. This led to a 6.5% withdrawal rate specifically due to injection site reactions in our AUD trial compared with only 0.5% in exenatide-treated patients with type 2 diabetes (55). The GI side effects, which are well recognized but typically transient (56), were in the exenatide group (44.1%) higher than reported in diabetes trials (57, 58). Also, 23.6% of placebo-treated patients experienced GI side effects, indicating that this group of patients may have a GI vulnerability (59). Only a single RCT has investigated the effects of pretreatment with antiemetics, reporting a significant reduction in nausea and vomiting in exenatide-treated healthy subjects (60).

Large dropout rates are often observed in AUD intervention trials (61), and the present study — with 54.3% dropout — is no exception. Although our sensitivity analysis (Supplemental Table 9) confirmed the robustness of the results even with imputations of missing data, the present dropout rate (69 of 127) remains a concern in evaluating the reproducibility and reliability of the findings. Weekly visits for 26 weeks might have been a contributory factor. However, in accordance with the EMA guidelines (62), we chose a study duration of 26 weeks to see whether there was a sustained treatment effect, lasting longer than the 12 weeks often reported for alcohol RCTs (63).

The approved 2 mg dosing regimen for diabetes patients is reported as the maximally efficacious dose for glucose control, reduction in body weight, and tolerable side effects (64). Our data also show that AUD patients obtain the same incretin response as diabetes patients with respect to improved glycemic control, weight loss, and side effects. Also preclinically, the standard exenatide dose used in preclinical food reward trials (65) has shown effects in preclinical alcohol self-administration experiments (66, 67). We did report a central effect in the brain imaging substudies, but of course we cannot rule out that the standard dose given was too low to elicit a reduction in number of heavy drinking days. However, the mean plasma exenatide level in this study was 4 times as high as that reported as the minimal effective concentration in humans, about 50 pg/mL (68). Also, because of safety concerns in this vulnerable group of patients, we did not raise the dose above the registered dose for treatment of type 2 diabetes.

Previous studies in diabetes patients have reported that while 45% of individuals receiving exenatide generate low-titer anti-exenatide antibodies (69), there is no apparent correlation between antibody titers and the effect of exenatide on mean HbA1c (57, 69). To the best of our knowledge, there is also no evidence of altered exenatide clearance in AUD patients. The renal elimination of exenatide (5) is an advantage in this group of patients, who typically have a heightened risk of hepatic injury (1).

One would expect a correlation between reduced brain alcohol cue reactivity and alcohol consumption. However, this was not the case in the present study, neither for the whole group of patients (*n* = 127) nor for the subgroup of patients that were fMRI-scanned (*n* = 22) or SPECT-scanned (*n* = 16). The sample size of the fMRI BMI subgroups with BMI less than 25 (*n* = 7) or BMI greater than 30 (*n* = 5) was too small to further explore whether the overall fMRI striatal responses were correlated with heavy drinking days in the overweight or obese subgroups. Only a few RCTs on AUD patients including fMRI measurements at baseline and follow-up have been performed (70), and most studies have been underpowered or had too much variation in study populations to report significant clinical treatment effects (71).

## Methods

### Trial design.

This randomized, placebo-controlled, double-blinded clinical trial was conducted at 4 alcohol outpatient clinics in Copenhagen, Denmark. The main trial comprised a 26-week treatment period investigating the primary and secondary endpoints. To evaluate the potential long-term effects, a single follow-up visit was conducted 6 months after treatment (24). A subgroup of the participants also underwent an fMRI scan and a single-photon emission CT (SPECT) DAT scan at baseline and after 26 weeks of treatment.

### Patients.

All potential participants received oral and written information about the project. Before signing of the written consent form, the alcohol breath concentration had to be below 0.5‰, which is the same limit as for driving a motor vehicle in Denmark (72). Eligible patients were 18–70 years of age, diagnosed with AUD according to DSM-5 and alcohol dependence according to ICD-10, and seeking treatment. Inclusion criteria required a minimum of 5 heavy drinking days, i.e., 60/48 g (men/women) of alcohol or more per day, in the past 30 days, measured by the Time-Line Follow Back (TLFB) method (73). Key exclusion criteria included severe mental disorder, other drug use disorder, a history of diabetes, pancreatitis, alcohol withdrawal seizures, and current treatment with drugs against alcohol dependence (disulfiram, acamprosate, naltrexone, and nalmefene). Full inclusion and exclusion criteria are listed in Supplemental Table 10. The healthy controls included in the fMRI substudy (*n* = 25) were matched by sex, age, and educational level. All patients were recruited from outpatient alcohol treatment facilities in the suburbs of Copenhagen or through our project webpage, and healthy controls via the project webpage. No patients were involved in setting the research question, planning the study, or interpreting or writing up the results. The results of the trial and the assigned intervention will be disseminated to all patients and healthy participants.

### Procedures.

The randomization was stratified in terms of sex, age (+/– 40 years of age), and number of heavy drinking days at baseline (4 strata), and the patients were randomly assigned 1:1 by Research Electronic Data Capture (REDCap) (74) to receive 2 mg exenatide once weekly (Bydureon) or placebo subcutaneously. The weekly injections were administered by an unblinded project nurse who did not participate in any assessments or behavioral treatment sessions. No randomization was performed in the imaging subgroup, as all eligible patients were invited to participate.

Patients who participated in the brain imaging substudy were scanned before receiving the first injection and again after 26 weeks of treatment. Throughout the trial, patients received the assigned treatment while wearing blindfolds by an unblinded nurse at the outpatient clinic, to whom they also delivered their weekly alcohol diary. Patients were assessed by blinded project staff at the time of screening, at weeks 4, 12, 20, and 26 (end of the main trial), and at the long-term 6-month follow-up visit (Supplemental Table 11 and Supplemental Figure 2). At every assessment, weight and somatic symptoms or diseases since the last visit were recorded, and safety blood samples were collected. In case medical assistance was needed, a 24-hour phone line was available. As a safety precaution due to earlier reports of pancreatitis caused by GLP-1 receptor agonist treatment (75), blood pancreas amylase was measured at all assessments. Participants with initial severe GI side effects received injections every second week for the first 6 weeks to reduce GI symptoms. All harms were recorded up until 10 weeks after termination of the intervention — i.e., week 26.

Throughout the trial, all patients received the assigned treatment as an add-on to standard AUD behavioral treatment, which included therapy sessions every second week, with a combination of motivational interviewing, cognitive therapy, and family therapy with a blinded therapist. Patients discontinuing the trial after a minimum of 8 weeks were encouraged to participate in a premature final visit and rescan. Only patients completing the week 26 visit (premature + per protocol) were invited for the long-term 6-month follow-up visit.

The healthy fMRI control group was assessed for eligibility before brain imaging at the Neurobiology Research Unit at Rigshospitalet, Copenhagen, Denmark. See Supplemental Appendices 1 and 2 for full details of the fMRI and SPECT substudies, respectively.

### Outcomes.

The primary endpoint was change in heavy drinking days, from baseline to week 26, as recorded by the TLFB method. Secondary endpoints included changes in total alcohol consumption; number of days with no alcohol consumption; Penn Alcohol Craving Scale score; Alcohol Use Disorders Identification Test (AUDIT) score; Drug Use Disorders Identification Test (DUDIT) score; Screen for Cognitive Impairment in Psychiatry (SCIP) test; Fagerström Test for Nicotine Dependence; blood γ-glutamyl transferase; blood alanine aminotransferase; blood phosphatidylethanol (PEth); mean cell volume; glycemic control parameters (HbA1c); body weight; blood pressure; heart rate; measures of health and life quality, i.e., 36-Item Short Form Health Survey (SF-36) and Symptom Checklist-92 (SCL-92); SPECT DAT specific binding ratio (BP_ND_); blood oxygen level–dependent (BOLD) fMRI signal change; change in subjective craving assessed with an alcohol cue reactivity task; change in top-down regulation assessed with an fMRI spatial working memory task; and change in heavy drinking days at 6-month follow-up. Additional methodological details regarding the analysis of blood and urine samples are given in Supplemental Methods.

### Data availability.

The study protocol, statistical analysis plan, and deidentified individual participant data, except raw fMRI and SPECT data and alcohol diaries, are available at the Mendeley database (76). Criteria for access to data are a methodologically sound proposal with an approved aim directed to the corresponding author, and requestors will have to sign a data access agreement. Data will be available for 5 years.

### Statistics.

The study was designed to have 90% power to detect a 28–percentage point treatment difference between the 2 groups with an estimated dropout of 40%. We planned to include 114 patients, but owing to a 60% dropout, we extended enrollment until October 1, 2019, or until 144 patients were included, whichever came first. All continuous outcomes were analyzed with an ANOVA adjusted for baseline until the last observational endpoint, and missing data were imputed with the use of multiple imputations in the mice package (77) in R software version 3.6.0 (78), method = pmm (predictive mean matching), and the number of imputed data sets = 100.

No adjustment for covariates was performed. SCIP data were analyzed with a linear mixed model, adjusted for benzodiazepine intake at the time of the assessment. DUDIT data were analyzed with a censored regression model due to zero-inflated values. An exploratory subgroup analysis based on the WHO BMI categories (79) was performed to see whether the effect of the treatment was related to baseline BMI. The statistical analysis plan was uploaded to the ClinicalTrials.gov homepage (80), and the data set was locked before any analyses were performed. All statistical analyses, except the post hoc analysis regarding exenatide plasma levels, were performed blinded. The hypothesis test was 2-sided, the level of statistical significance was 5%, and a confidence interval of 95% was used. All efficacy and safety analyses were performed according to the intention-to-treat principle. Analyses were performed with R software version 3.6.0 (78). See Supplemental Appendices 1 and 2 for the complete statistical method for the fMRI and SPECT analyses.

### Study approval.

The protocol was approved by the Danish Ethics Committee of the Capital Region, Copenhagen, Denmark (H-17003043), the Danish Medical Agency (2017014028), and the Danish Data Protection Agency (RHP-2017-029). The trial was monitored by an independent study monitor (Good Clinical Practice unit, Copenhagen, Denmark). Protocol modifications performed after trial commencement are shown in Supplemental Table 12. All participants provided written informed consent prior to study inclusion. The funding sources and the manufacturer of exenatide once weekly (Bydureon, AstraZeneca) had no influence on the trial design or data analysis. The trial was conducted according to the Declaration of Helsinki, and the authors assume responsibility for the accuracy of data, analysis, and overall fidelity to the trial protocol. Trial registrations: ClinicalTrials.gov, NCT03232112; EudraCT: 2016-003343-11.

## Author contributions

Conceptualization was contributed by AFJ and TV. Data curation was performed by MKK and CTE. Statistical power analysis and statistical analysis plan was performed by MKK, AFJ and CTE. Clinical data were analyzed by MKK and CTE. SPECT data were analyzed by MKK and MEJ. fMRI N-back task were analyzed by JM. fMRI ALCUE data were analyzed by PMF, MKK, AL, and SVK. Plasma FGF-21 were analyzed and validated by MPG. Urine oxidative stress parameters were analyzed and validated by HEP. Plasma PINP, CTX, TRAP-5b were analyzed and validated by NRJ. Exenatide and antibody plasma levels were analyzed and validated by JJH. Plasma PEth levels were analyzed and validated by MLB. Funding acquisition were performed by AFJ and MKK. Clinical investigations including fMRI scans were performed by: MKK, MEJ, NLD, MM, CFJ, AMØJ, and VAZ. SPECT scans were performed by GKT. Methodology were planned by AFJ, TV, MKK, MEJ, KWM, HB, NDV, GMK, and UB. The project was administered by AFJ (sponsor investigator), MKK, and MEJ. fMRI ALCUE paradigme were provided by SVK. fMRI ALCUE adaptation to E-prime software were performed by PMF. Validation of clinical data were performed by MKK, AFJ, and CTE. Validation of fMRI ALCUE data were performed by MKK, AL, SVK, and PMF. Validation of fMRI N-back task data were performed by MKK and JM. Validation of SPECT data were performed by MKK, MEJ, and GKT. Visualization of clinical data were performed by MKK, MEJ, and AFJ. Visualization of SPECT data were performed by MKK, MEJ, and AFJ. Visualization of fMRI N-back task data data were performed by JM. Visualization of fMRI ALCUE data were performed by SVK, MKK, AL, MEJ, and AFJ. Writing of the draft of the fMRI N-back task results and analysis were performed by JM. Writing of the original manuscript draft was performed by MKK. All authors have contributed to the review and editing of the manuscript.

## Supplementary Material

Supplemental data

ICMJE disclosure forms

## Figures and Tables

**Table 1:**
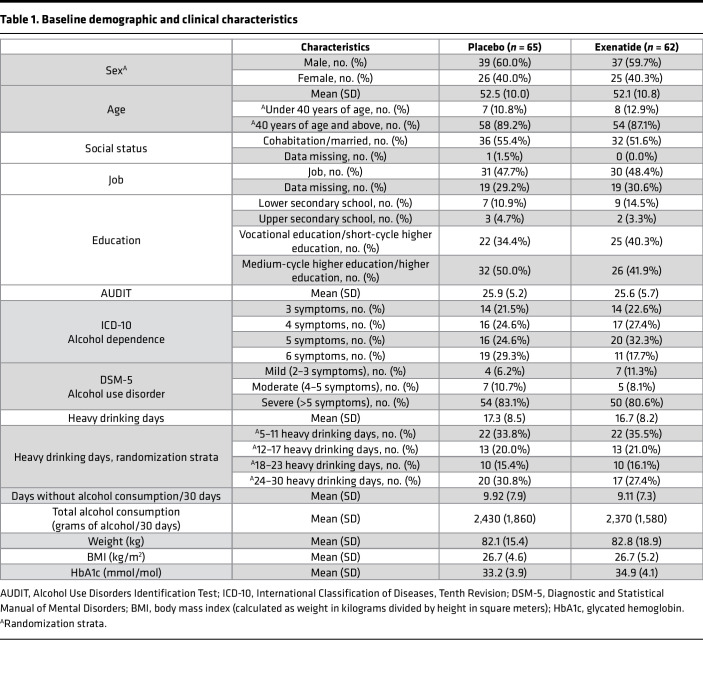
Baseline demographic and clinical characteristics

**Table 2:**
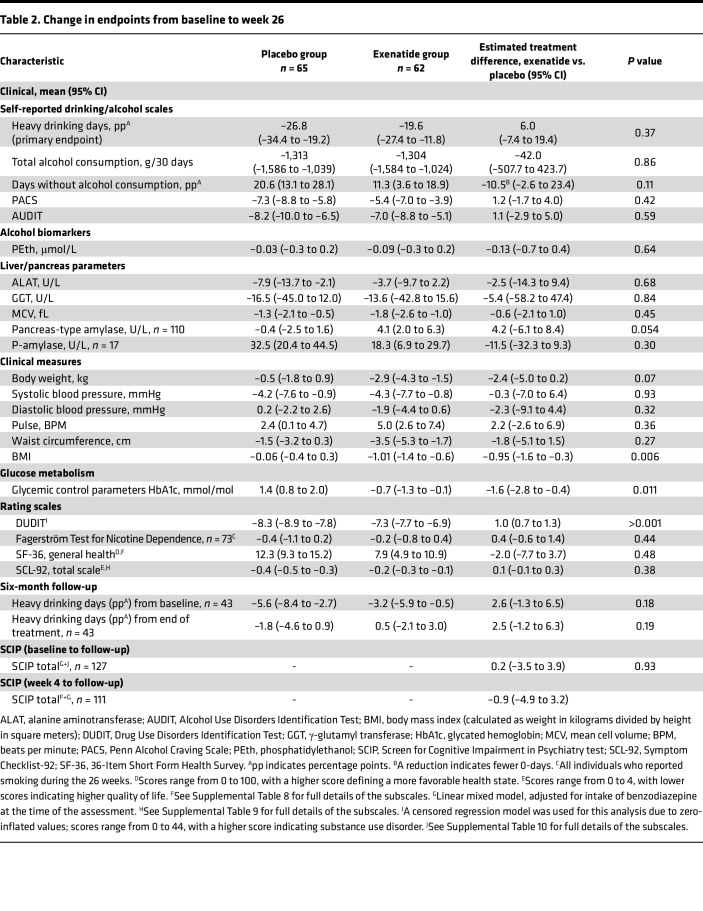
Change in endpoints from baseline to week 26

**Table 3:**
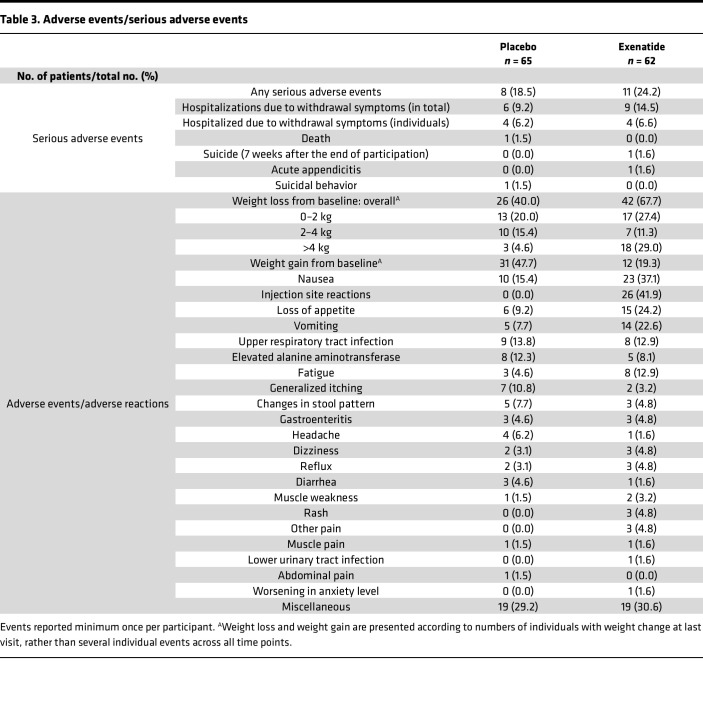
Adverse events/serious adverse events

## Version 1 09/06/2022

In-Press Preview

## Version 2 10/10/2022

Electronic publication

## References

[1] Carvalho AF (2019). Alcohol use disorders. Lancet.

[2] Askgaard G (2020). Hospital admissions and mortality in the 15 years after a first-time hospital contact with an alcohol problem: a prospective cohort study using the entire Danish population. Int J Epidemiol.

[3] Kranzler HR, Soyka M (2018). Diagnosis and pharmacotherapy of alcohol use disorder: a review. JAMA.

[4] Bottlender M, Soyka M (2005). Outpatient alcoholism treatment: predictors of outcome after 3 years. Drug Alcohol Depend.

[5] Holst JJ (2007). The physiology of glucagon-like peptide 1. Physiol Rev.

[6] Reddy IA (2016). Glucagon-like peptide 1 receptor activation regulates cocaine actions and dopamine homeostasis in the lateral septum by decreasing arachidonic acid levels. Transl Psychiatry.

[7] Nuffer WA, Trujillo JM (2015). Liraglutide: a new option for the treatment of obesity. Pharmacotherapy.

[8] Volkow ND (2013). Obesity and addiction: neurobiological overlaps. Obes Rev.

[9] Cork SC (2015). Distribution and characterisation of glucagon-like peptide-1 receptor expressing cells in the mouse brain. Mol Metab.

[10] Han VKM (1986). Cellular localization of proglucagon/glucagon-like peptide I messenger RNAs in rat brain. J Neurosci Res.

[11] Jensen CB (2018). Characterization of the glucagonlike peptide-1 receptor in male mouse brain using a novel antibody and in situ hybridization. Endocrinology.

[12] Merchenthaler I (1999). Distribution of pre-pro-glucagon and glucagon-like peptide-1 receptor messenger RNAs in the rat central nervous system. J Comp Neurol.

[13] Rinaman L (2010). Ascending projections from the caudal visceral nucleus of the solitary tract to brain regions involved in food intake and energy expenditure. Brain Res.

[14] Vrang N, Grove K (2011). The brainstem preproglucagon system in a non-human primate (Macaca mulatta). Brain Res.

[15] Olds J, Milner P (1954). Positive reinforcement produced by electrical stimulation of septal area and other regions of rat brain. J Comp Physiol Psychol.

[16] Heppner KM (2015). Expression and distribution of glucagon-like peptide-1 receptor mRNA, protein and binding in the male nonhuman primate (Macaca mulatta) brain. Endocrinology.

[17] Thomsen M (2018). Effects of glucagon-like peptide 1 analogs on alcohol intake in alcohol-preferring vervet monkeys. Psychopharmacology (Berl).

[18] Brunchmann A (2019). The effect of glucagon-like peptide-1 (GLP-1) receptor agonists on substance use disorder (SUD)-related behavioural effects of drugs and alcohol: a systematic review. Physiol Behav.

[19] Suchankova P (2015). The glucagon-like peptide-1 receptor as a potential treatment target in alcohol use disorder: evidence from human genetic association studies and a mouse model of alcohol dependence. Transl Psychiatry.

[20] Athauda D (2017). Exenatide once weekly versus placebo in Parkinson’s disease: a randomised, double-blind, placebo-controlled trial. Lancet.

[21] Yammine L (2021). Exenatide adjunct to nicotine patch facilitates smoking cessation and may reduce post-cessation weight gain: a pilot randomized controlled trial. Nicotine Tob Res.

[22] Klausen MK (2021). The role of glucagon-like peptide 1 (GLP-1) in addictive disorders. Br J Pharmacol.

[23] Sobell MB (1986). The reliability of a timeline method for assessing normal drinker college students’ recent drinking history: utility for alcohol research. Addict Behav.

[24] Antonsen KK (2018). Does glucagon-like peptide-1 (GLP-1) receptor agonist stimulation reduce alcohol intake in patients with alcohol dependence: study protocol of a randomised, double-blinded, placebo-controlled clinical trial. BMJ Open.

[25] Yaribeygi H (2021). GLP-1 mimetics and cognition. Life Sci.

[26] Nutt DJ (2015). The dopamine theory of addiction: 40 years of highs and lows. Nat Rev Neurosci.

[27] Volkow ND (2002). Role of dopamine, the frontal cortex and memory circuits in drug addiction: insight from imaging studies. Neurobiol Learn Mem.

[28] Witkiewitz K (2017). Clinical validation of reduced alcohol consumption after treatment for alcohol dependence using the World Health Organization risk drinking levels. Alcohol Clin Exp Res.

[29] Maldjian JA (2003). An automated method for neuroanatomic and cytoarchitectonic atlas-based interrogation of fMRI data sets. Neuroimage.

[30] Vallöf D (2016). The glucagon-like peptide 1 receptor agonist liraglutide attenuates the reinforcing properties of alcohol in rodents. Addict Biol.

[31] Shirazi RH (2013). Gut peptide GLP-1 and its analogue, Exendin-4, decrease alcohol intake and reward. PLoS One.

[32] Anton RF (2006). Combined pharmacotherapies and behavioral interventions for alcohol dependence: the COMBINE study: a randomized controlled trial. JAMA.

[33] Johnson BA (2003). Oral topiramate for treatment of alcohol dependence: a randomised controlled trial. Lancet.

[34] Carroll KM (2017). Cognitive Behavioral Interventions for Alcohol and Drug Use Disorders: Through the Stage Model and Back Again. Psychol Addict Behav.

[35] Scherrer B (2021). Baseline severity and the prediction of placebo response in clinical trials for alcohol dependence: a meta-regression analysis to develop an enrichment strategy. Alcohol Clin Exp Res.

[36] Litten RZ (2013). The placebo effect in clinical trials for alcohol dependence: an exploratory analysis of 51 naltrexone and acamprosate studies. Alcohol Clin Exp Res.

[37] Butler T (2014). Comparison of human septal nuclei MRI measurements using automated segmentation and a new manual protocol based on histology. Neuroimage.

[38] Bruijnen CJWH (2019). Prevalence of cognitive impairment in patients with substance use disorder. Drug Alcohol Rev.

[39] Wilcox CE (2014). Cognitive control in alcohol use disorder: deficits and clinical relevance. Rev Neurosci.

[40] Petersen CS, Miskowiak KW (2020). Toward a transdiagnostic neurocircuitry-based biomarker model for pro-cognitive effects: challenges, opportunities, and next steps. CNS Spectr.

[41] Volkow ND (1996). Decreases in dopamine receptors but not in dopamine transporters in alcoholics. Alcohol Clin Exp Res.

[42] Yen CH (2015). Reduced dopamine transporter availability and neurocognitive deficits in male patients with alcohol dependence. PLoS One.

[43] Volkow ND (2007). Profound decreases in dopamine release in striatum in detoxified alcoholics: possible orbitofrontal involvement. J Neurosci.

[44] Jensen ME (2020). Glucagon-like peptide-1 receptor regulation of basal dopamine transporter activity is species-dependent. Neurochem Int.

[45] Anandhakrishnan A, Korbonits M (2016). Glucagon-like peptide 1 in the pathophysiology and pharmacotherapy of clinical obesity. World J Diabetes.

[46] Eldor R (2016). Discordance between central (brain) and pancreatic action of exenatide in lean and obese subjects. Diabetes Care.

[47] Edwards CMB (2001). Exendin-4 reduces fasting and postprandial glucose and decreases energy intake in healthy volunteers. Am J Physiol Endocrinol Metab.

[48] Biery JR (1991). Alcohol craving in rehabilitation: assessment of nutrition therapy. J Am Diet Assoc.

[49] Johansen NJ (2020). Effect of short-acting exenatide administered three times daily on markers of cardiovascular disease in type 1 diabetes: a randomized double-blind placebo-controlled trial. Diabetes Obes Metab.

[50] Skytte MJ (2020). Effects of a highly controlled carbohydrate-reduced high-protein diet on markers of oxidatively generated nucleic acid modifications and inflammation in weight stable participants with type 2 diabetes; a randomized controlled trial. Scand J Clin Lab Invest.

[51] Kjær LK (2017). Cardiovascular and all-cause mortality risk associated with urinary excretion of 8-oxoguo, a biomarker for RNA oxidation, in patients with type 2 diabetes: a prospective cohort study. Diabetes Care.

[52] Mabilleau G (2018). Novel skeletal effects of glucagon-like peptide-1 (GLP-1) receptor agonists. J Endocrinol.

[53] Storgaard H (2017). Glucagon-like peptide-1 receptor agonists and risk of acute pancreatitis in patients with type 2 diabetes. Diabetes Obes Metab.

[54] Sattar N (2021). Cardiovascular, mortality, and kidney outcomes with GLP-1 receptor agonists in patients with type 2 diabetes: a systematic review and meta-analysis of randomised trials. Lancet Diabetes Endocrinol.

[55] Jones SC (2015). Injection-site nodules associated with the use of exenatide extended-release reported to the U.S. Food and Drug Administration Adverse Event Reporting System. Diabetes Spectr.

[56] Trujillo J (2020). Safety and tolerability of once-weekly GLP-1 receptor agonists in type 2 diabetes. J Clin Pharm Ther.

[57] Drucker DJ (2008). Exenatide once weekly versus twice daily for the treatment of type 2 diabetes: a randomised, open-label, non-inferiority study. Lancet.

[58] MacConell L (2015). Safety and tolerability of exenatide once weekly in patients with type 2 diabetes: an integrated analysis of 4,328 patients. Diabetes Metab Syndr Obes.

[59] Haber PS, Kortt NC (2021). Alcohol use disorder and the gut. Addiction.

[60] Ellero C (2010). Prophylactic use of anti-emetic medications reduced nausea and vomiting associated with exenatide treatment: a retrospective analysis of an open-label, parallel-group, single-dose study in healthy subjects. Diabet Med.

[61] Hallgren KA, Witkiewitz K (2013). Missing data in alcohol clinical trials: a comparison of methods. Alcohol Clin Exp Res.

[63] Wallach JD (2020). Characteristics of ongoing clinical trials for alcohol use disorder registered on ClinicalTrials.gov. JAMA Psychiatry.

[64] Fineman M (2011). Pharmacokinetics and pharmacodynamics of exenatide extended-release after single and multiple dosing. Clin Pharmacokinet.

[65] Dickson SL (2012). The glucagon-like peptide 1 (GLP-1) analogue, exendin-4, decreases the rewarding value of food: a new role for mesolimbic GLP-1 receptors. J Neurosci.

[66] Thomsen M (2017). The glucagon-like peptide 1 receptor agonist Exendin-4 decreases relapse-like drinking in socially housed mice. Pharmacol Biochem Behav.

[67] Egecioglu E (2013). The glucagon-like peptide 1 analogue Exendin-4 attenuates alcohol mediated behaviors in rodents. Psychoneuroendocrinology.

[68] Taylor K (2005). Day-long subcutaneous infusion of exenatide lowers glycemia in patients with type 2 diabetes. Horm Metab Res.

[69] Fineman MS (2012). Clinical relevance of anti-exenatide antibodies: safety, efficacy and cross-reactivity with long-term treatment. Diabetes Obes Metab.

[70] Schacht J (2013). Functional neuroimaging studies of alcohol cue reactivity: a quantitative meta-analysis and systematic review. Addict Biol.

[71] Courtney KE (2016). Neural substrates of cue reactivity: association with treatment outcomes and relapse. Addict Biol.

[72] Bernhoft IM, Behrensdorff I (2003). Effect of lowering the alcohol limit in Denmark. Accid Anal Prev.

[74] Harris PA (2019). The REDCap Consortium: building an international community of software platform partners. J Biomed Inform.

[75] Elashoff M (2011). Pancreatitis, pancreatic, and thyroid cancer with glucagon-like peptide-1-based therapies. Gastroenterology.

[76] https://data.mendeley.com/datasets/hck4tvrhbb.

[77] van Buuren S, Groothuis-Oudshoorn K (2011). mice: multivariate imputation by chained equations in R. J Stat Softw.

[78] https://www.R-project.org/.

[79] World Health Organization (2000). Obesity: preventing and managing the global epidemic. Report of a WHO consultation. World Health Organ Tech Rep Ser.

[80] https://clinicaltrials.gov/ct2/show/NCT03232112?term=NCT03232112&draw=2&rank=1.

