# Supplementary material for: Exenatide once weekly for alcohol use disorder investigated in a randomized, placebo-controlled clinical trial
Source: JCI Insight. 2022 Oct 10;7(19):e159863. doi: 10.1172/jci.insight.159863 (PMC9675448; doi:10.1172/jci.insight.159863)
Supplement: Supplemental data [file jciinsight-7-159863-s185.pdf]

Supplementary Materials for

Exenatide once weekly for alcohol use disorder - a randomized, placebo-controlled clinical trial

Mette Kruse Klausen, Mathias Ebbesen Jensen, Marco Møller, Nina Le Dous, Anne-Marie Østergaard Jensen, Victoria Alberte Zeeman, Claas-Frederik Johannsen, Alycia Lee, Gerda Krog Thomsen, Julian Macoveanu, Patrick MacDonald Fisher, Matthew Paul Gillum, Niklas Rye Jørgensen, Marianne Lerbæk Bergmann, Henrik Enghusen Poulsen, Ulrik Becker, Jens Juul Holst, Helene Benveniste, Nora D. Volkow, Sabine Vollstädt-Klein, Kamilla Woznika Miskowiak, Claus Thorn Ekstrøm, Gitte Moos Knudsen, Tina Vilsbøll, Anders Fink-Jensen*

*Correspondence to: anders.fink-jensen@regionh.dk

This PDF file includes:

Analysis of serum, plasma, and urine samples

Supplemental Figure 1 to 3

Supplemental Table 1 to 12

Appendix 1: fMRI Alcohol cue-reactivity (ALCUE) and Spatial working memory (N-back task)

Appendix 2: SPECT (single photon emission computed tomography scan)

Analysis of serum, plasma, and urine samples

All samples were collected at variable time points during the day without an overnight fast.

Blood samples for serum were collected in a Vacuette Serum 9 ml tube and stored at room temperature for 1-2 hours before 15 minutes of centrifugation at 4°C/39.2°F, and 2.614 RPM/1.100 RCF. Blood samples for plasma were collected in a Vacuette K2 EDTA 9 ml tube and kept on ice for a maximum of 30 minutes before 15 minutes of centrifugation at 4°C/39.2°F, and 2.614 RPM/1.100 RCF. All samples were immediately stored at -20°C/-4°F and within two months transferred securely to -80 °C/-112°F, where they were kept for a maximum of three years, before being analyzed. An additional Vacuette K2 EDTA 9mL tube, and 4 mL of urine collected at baseline and week 26, was stored for a future biobank.

Phosphatidylethanol (PEth)

PEth samples were collected in a Vacuette K2 EDTA 2 ml tube and immediately transferred to a freezer as described above. Sample preparation and analysis: using a Hamilton STARlet workstation, 200 µL whole blood was precipitated with 800 µL isopropanol, with d5-Peth (16:0/18:1) as internal standard, in a 96-well microtiter plate. The plate was centrifuged, and the supernatant analysed on a Waters Acquity ultra-performance liquid chromatograph (UPLC) with Xevo TQ-S tandem mass spectrometer operated in electrospray negative mode. The chromatographic separation was achieved with a Phenomenex Kinetex XB-C18 (30 x 2.1 mm, 2.6 µm) column. The multiple reaction monitoring (MRM) transitions used for Peth (16:0/18:1) was 701.46 >281.07 as quantifier and 701.46>255.26 as a qualifier. For the internal standard d5-Peth (16:0/18:1) 706.46 > 281.07 was used.

Fibroblast growth factor 21 (FGF-21)

Intact (i.e., full-length and active) plasma FGF21 was analyzed by ELISA using detection and capture antibodies targeted to the N and C-termini of the full-length human protein (EagleBiosciences, Nashua NH, USA Cat#: F21K31-K01) (1).

One participant was excluded as an outlier from the analysis due to an extreme follow-up value of 19000 pg/mL, probably caused by a breath alcohol level of 2.47 ‰ at the assessment time.

Bone markers (CTX, TRAcP-5b, P1NP)

Plasma CTX was measured using the IDS-iSYS CTX (CrossLaps®) assay (Immunodiagnostic Systems, plc, Tyne and Wear, UK). Plasma PINP was measured using the IDS-iSYS intact PINP assay (Immunodiagnostic Systems). Tartrate-resistant acid phosphatase 5b (TRAcP5b) was measured using the BoneTRAP® assay (Immunodiagnostic Systems). All assays were carried out on a dedicated automated analyzer, iSYS (Immunodiagnostic Systems), according to the manufacturer's instructions. All assays are chemiluminescence immunoassays.

All analyses were done with plasma as the sample material. For each assay the sample aliquots were kept frozen at - 80 degrees Celsius until the day of analysis. None of the samples had previously been thawed, and all analyses were performed immediately after thawing the samples. All samples were analyzed using one single batch of each assay. The intermediary precisions expressed as coefficients of variation for CTX were 5.3% (at CTX concentration 213 ng/L), 3.4% (869 ng/L), and 3.5% (2,113 ng/L) for iSYS. For P1NP the intermediary precisions were 5.4% (18.96 µg/L), 6.5% (48.48 µg/L), and 6.1% (122.10 µg/L) for iSYS. For TRAcP5b the intermediary precisions were 10.9% (3.2 U/L), 4.8% (6.2 U/L), and 5.4% (9.0 U/L).

A total of 13 participants were excluded from the final analysis due to alterations in medications known to affect bone mass (thiazides: 7 patients; thyroid-hormone replacement: 1 patient; oral contraceptives: 3 patients; bisphosphonates: 2 patients), leaving 114 patients (placebo group: 58, exenatide group: 55) to be included in the final analysis.

The lowest detectable value for TRAcP-5b is 0.9 U/L. This value was replaced with 0.9/2= 0.45 (17 samples).

Exenatide

Plasma samples for analyses of exenatide, anti-exenatide antibodies, and glucagon were stored at −80°C as previously described. Exenatide levels were measured by radioimmunoassay. The sensitivity of the exenatide assay is <1 pmol/L, but to avoid plasma interference, samples were diluted 10-fold in assay buffer. To estimate antibodies against exenatide, plasma samples were incubated with 125-I-labelled exendin-4, which binds to the antiserum with the same energy as full-length exenatide tracer-antibody complexes separated from the mixture using plasma-coated charcoal as in the exenatide radioimmunoassay. Any increases in the binding of the tracer above that observed in plasma from subjects never exposed to exenatide indicate the presence of

antibodies. Results are presented in percent binding of the tracer, a proxy of antibody titer. The glucagon assay employs a C-terminally directed antiserum and therefore measures glucagon of mainly pancreatic origin. Sensitivity was <1 pmol/L. The detection level was 40 pmol/l for exenatide and 4% for the anti-exenatide antibodies.

Two patients were excluded from the analysis due to baseline antibodies >35.

Urine oxidative stress markers

All samples were collected at variable time points throughout the day. The urine samples were stored at -20°C until analysis for oxidative stress markers (8-oxo-7,8-dihydroguanosine (8-oxoGuo) and 8-oxo-7,8-dihydro-2'-deoxyguanosine (8-oxodG)) and were analyzed using an ultra-performance liquid chromatography-tandem mass spectrometry (UPLC-MS/MS) system as previously described (2). Urinary excretion of 8-oxoGuo and 8-oxodG was normalized to urine creatinine.

Figure 1: Flowchart 6-month follow-up

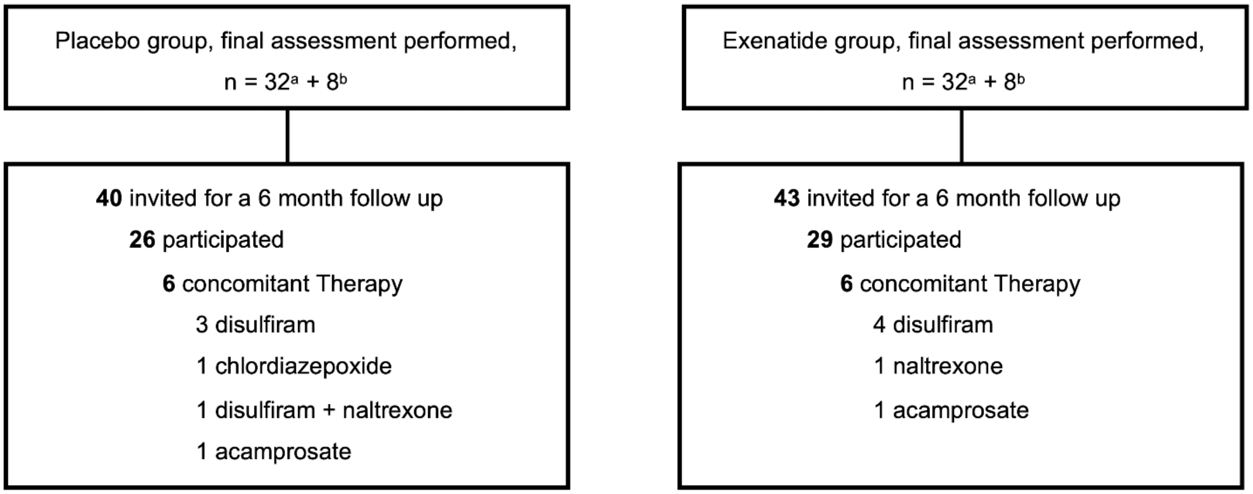

[a]finished per protocol, [b]finished prematurely. Twelve individuals were excluded from the analyses (except for the Fagertröms Test for Nicotine Dependence) due to concomitant therapy at the assessment time

Table S1: Weeks with study medication

Distribution of injections in the main treatment period (26 weeks)

Treatment group	Patients	Summary statistics	Injections	% of injections*
Placebo	32	Mean (SD)	22.1(2.75)	85.0 (10.6)
Exenatide	26	Mean (SD)	22.6(2.23)	87.0 (8.57)
Overall	58	Mean (SD)	22.3 (2.52)	85.9 (9.70)

Only patients who completed the full intervention (26 weeks) were included (n=58) *distribution of the individual patient percentages of study injections

Table 2: Pre-specified post hoc analysis

Urine oxidative stress/bone-markers/FGF-21/exenatide

Change in endpoints from baseline to week 26

**Characteristic**	**Placebo group n = 65**	**Exenatide group n = 62**	**Estimated treatment difference, exenatide vs. placebo (95% Cl)**	**p - value**
**Clinical, mean (95%Cl)**				
Urine 8-oxoGuo (nmol/mmol creatinine)[a]	-0.17 (-0.28 to -0.07)	0.22 (0.11 to 0.32)	0.24 (0.04 to 0.44)	0.022
urine-8oxodG (nmol/mmol creatinine)[a]	-0.24 (-0.38 to -0.09)	0.30 (0.15 to 0.45)	0.43 (0.15 to 0.72)	0.003
Plasma-TRAcP-5b[b] (n=114) (U/L)	0.13 (-0.14 to 0.40)	0.25 (-0.02 to 0.53)	0.10 (-0.53 to 0.74)	0.74
Plasma-CTX[b] (n=114) (ng/L)	73.3 (22.6 to 124.0)	77.9 (26.2 to 129.5)	7.3 (-97.5 to 112.1)	0.89
Plasma-P1NP[b] (n=114) (µg/L)	8.0 (2.9 to 13.0)	3.1 (-2.1 to 8.2)	-3.6 (-13.5 to 6.3)	0.47
FGF-21 (pg/mL)[c]	-107.8 (-285.8 to 70.2)	-34.0 (-214.9 to 146.8)	-207.4 (-642.7 to 227.9)	0.35
Plasma exenatide (pmol/L)	3.2 (-12.9 to 19.2)	48.9 (34.1 to 63.7)	45.6 (16.5 to 74.7)	0.003
Overall anti-exenatide antibody binding (%)	0.6 (-3.8 to 5.0)	16.6 (12.6 to 20.7)	16.1 (6.9 to 25.3)	0.002

[a]Urine oxidative stress markers, 8-oxoGUO = 8-oxo-7,8-dihydroguanosine, baseline mean =2.28 nmol/mmol creatinine and 8-oxodG = 8-Oxo-2'-deoxyguanosine, baseline mean = 1.73 nmol/mmol creatinine; [b]Bone marker, CTX = C-terminal telopeptide of type I collagen, PINP = N-terminal propeptide of type I procollagen, TRAP-5B =Tartrate resistant acid phosphatase; [c]liver protein, FGF-21 = Fibroblast growth factor 21. SI conversion factors: To convert Plasma-TRAcP-5b levels to µkat/L, multiply by 0.0167. To convert plasma exenatide levels to pg/mL, multiply by 4.187.

Table 3: Short-Form Health Survey (SF-36)

Change in endpoints from baseline to week 26

Characteristic	Placebo group n = 65	Exenatide group n = 62	Estimated treatment difference, exenatide vs placebo (95% Cl)	p-value
**Clinical, mean (95%Cl)**				
Physical functioning	5.2 (2.7 to 7.7)	1.2 (-1.4 to 3.8)	-3.5 (-8.6 to 1.5)	0.17
Role limitations due to physical health	17.3 (9.9 to 24.8)	18.5 (10.9 to 26.1)	1.8 (-14.3 to 17.8)	0.83
Role limitations due to emotional problems	25.9 (18.8 to 33.1)	20.6 (13.3 to 27.9)	-2.6 (-16.6 to 11.5)	0.72
Energy/fatigue	12.4 (8.1 to 16.7)	11.0 (6.6 to 15.4)	-0.3 (-9.9 to 9.4)	0.96
Emotional well-being	12.2 (8.6 to 15.9)	10.1 (6.4 to 13.9)	-1.1 (-9.1 to 6.8)	0.78
Social functioning[a]	NA	NA	NA	NA
Pain	-4.1 (-8.5 to 0.3)	-7.4 (-11.9 to -2.9)	0.2 (-9.4 to 9.8)	0.97
General Health	12.3 (9.3 to 15.2)	7.9 (4.9 to 10.9)	-2.0 (-7.7 to 3.7)	0.48

Scores range from 0-100 with a high score defining a more favorable health state. Missing data (left blank) were not taken into account, and scale scores represent the average for all items in the scale answered (3).

[a]The results from this subscore, was by mistake wrongly recorded.

Table 4: Symptom Checklist (SCL-92)

Change in endpoints from baseline to week 26

Characteristic	Placebo group n = 65	Exenatide group n = 62	Estimated treatment difference, exenatide vs placebo (95% Cl)	p-value
**Clinical, mean (95%Cl)**				
Somatization	-0.11 (-0.23 to 0.004)	-0.11 (-0.23 to 0.007)	-0.07 (-0.31 to 0.17)	0.52
Anxiety-R	-0.46 (-0.55 to -0.37)	-0.3 (-0.39 to -0.20)	0.01 (-0.18 to 0.20)	0.92
Anxiety	-0.37 (-0.47 to -0.28)	-0.18 (-0.28 to -0.09)	0.08 (-0.09 to 0.24)	0.37
Interpersonal sensitivity	-0.45 (-0.54 to -0.36)	-0.21 (-0.30 to -0.12)	0.08 (-0.10 to 0.25)	0.38
Phobia	-0.31 (-0.19 to -0.43)	0.01 (-0.11 to 0.13)	0.16 (-0.08 to 0.40)	0.20
Obs-compulsive	-0.47 (-0.59 to -0.35)	-0.24 (-0.36 to -0.11)	0.10 (-0.15 to 0.34)	0.43
Depression	-0.59 (-0.72 to -0.46)	-0.41 (-0.54 to -0.28)	0.07 (-0.19 to 0.32)	0.61
Hostility	-0.10 (-0.20 to -0.01)	-0.15 (-0.25 to -0.31)	-0.08 (-0.25 to 0.09)	0.34
Paranoid	-0.38 (-0.45 to -0.32)	-0.15 (-0.21 to -0.08)	0.04 (-0.09 to 0.16)	0.55
Psychoticism	-0.22 (-0.28 to -0.17)	-0.12 (-0.17 to -0.06)	0.03 (-0.07 to 0.13)	0.55
Total scale	-0.37 (-0.46 to -0.28)	-0.16 (-0.25 to -0.06)	0.09 (-0.12 to 0.30)	0.38

Scores range from 0-4, with lower scores indicating higher quality of life. Missing data (left blank) were not taken into account, and scale scores represent the average for all items in the scale answered (3).

Table 5: Screen for Cognitive Impairment in Psychiatry test (SCIP)

All analysis has been adjusted for intake of benzodiazepine at the time of assessment. A higher score indicates an improvement in cognitive function.

Change in SCIP-score from baseline to week 26, n = 127

	**Estimated treatment difference, exenatide vs. placebo (95% Cl)**	**p-value**
SCIP 1 - Verbal Learning Test-Immediate (VLT-I)	-0.13 (-1.25 to 0.98)	0.81
SCIP 2 - Working Memory Test (WMT)	0.32 (-0.74 to 1.38)	0.55
SCIP 3 - Verbal Fluency Test (VFT)	0.10 (-1.47 to 1.66)	0.90
SCIP 4 - Verbal Learning Test-Delayed (VLT-D)	-0.53 (-1.19 to 0.13)	0.11
SCIP 5 - Processing Speed Test (PST)	0.39 (-0.38 to 1.14)	0.31
SCIP total	0.16 (-3.53 to 3.86)	0.93

Change in SCIP-score from week 4 to week 26, n = 111:

	**Estimated treatment difference, exenatide vs. placebo (95% Cl)**	**p-value**
SCIP 1 - Verbal Learning Test-Immediate (VLT-I)	-0.13 (-1.50 to 1.24)	0.85
SCIP 2 - Working Memory Test (WMT)	-0.25 (-1.52 to 1.02)	0.69
SCIP 3 - Verbal Fluency Test (VFT)	-0.06 (-1.84 to 1.72)	0.95
SCIP 4 - Verbal Learning Test-Delayed (VLT-D)	-0.56 (-1.30 to 0.17)	0.13
SCIP 5 - Processing Speed Test (PST)	0.10 (-0.82 to 1.02)	0.83
SCIP total	-0.87 (-4.94 to 3.20)	0.67

Table 6: Pre-specified subgroup analysis & post hoc WHO Drinking Risk Levels

Characteristic	Placebo group n = 65	Exenatide group n = 62	Estimated treatment difference, exenatide vs. placebo (95% Cl)	p-value
**Clinical, mean (95% Cl)**				
**Baseline heavy drinking days (randomisation strata)**				
5-11 heavy drinking days, n=44	-4.6 (-11.8 to 2.5)	-10.1 (-17.3 to -3.0)	-7.6 (-20.3 to 5.2)	0.25
12-17 heavy drinking days, n=26	-17.1 (-33.2 to -1.0)	-15.6 (-31.7 to 0.5)	3.1 (-24.2 to 30.3)	0.83
18-23 heavy drinking days, n=20	-40.4 (-58.4 to -22.3)	-49.4 (-67.5 to -31.4)	-4.8 (-37.9 to 28.3)	0.78
24-30 heavy drinking days, n=37	-65.0 (-80.9 to -49.1)	-45.9 (-63.1 to -28.6)	18.4 (-15.0 to 51.8)	0.29
**DSM-5 group**				
Mild: 2-3 symptoms, n=11	-8.9 (-28.4 to 10.6)	-20.3 (-35.1 to -5.6)	-1.0 (-27.9 to 25.9)	0.94
Moderate: 4-5 symptoms, n=12	-10.8 (-29.6 to 8.1)	-26.4 (-48.7 to -4.1)	-12.7 (-51.8 to 26.4)	0.55
Severe: >5 symptoms, n=104	-33.4 (-42.1 to -24.7)	-20.1 (-29.1 to -11.1)	10.6 (-4.8 to 26.0)	0.18
**Geography[b]**				
Hvidovre, n=36	-43.9 (-61.8 to -25.9)	-9.8 (-25.0 to 5.3)	26.8 (-2.1 to 55.7)	0.08
Lyngby, n=47	-30.1 (-40.5 to -19.73)	-33.4 (-44.3 to -22.6)	-5.6 (-23.3 to 12.2)	0.54
Glostrup, n=28	-23.1 (-32.8 to -13.5)	-23.8 (-36.8 to -10.8)	5.5 (-17.8 to 28.8)	0.65
Koege, n=17	-27.7 (-36.1 to -19.3)	-40.8 (-48.7 to -32.9)	8.4 (-6.3 to 23.1)	0.28
**Drinking Risk Levels**				
Reduction in WHO Drinking Risk Levels[c]	-1.3 (-1.6 to -1.1)	-1.4 (-1.6 to -1.1)	-0.04 (-0.6 to 0.5)	0.88

Pre-specified subgroup analyses & post hoc analysis, WHO Drinking Risk Levels. Change in heavy drinking days ([a]pp) from baseline to week 26. Abbreviations: DSM-5, Diagnostic and Statistical Manual of Mental Disorders; WHO, World Health Organization. [a]pp= percentage points, [b]suburbs of Copenhagen, Denmark, [c]points

Table 7: Exploratory BMI subgroup analysis

Change in heavy drinking days, and total alcohol intake from baseline to week 26

Characteristics	Placebo group	Exenatide group	Estimated treatment difference, exenatide vs placebo (95% Cl)	p-value
**Clinical, mean (95% Cl)**				
**Change in heavy drinking days (pp)[a]**				
Normal weight, n=52 (BMI = 18.5 - 24.9)	-46.1 (-58.4 to -33.7)	-12.3 (-23.3 to -1.3)	27.5 (4.7 to 50.2)	0.024
Pre-obesity, n=45 (BMI = 25.0-29.9)	-30.0 (-38.7 to -21.2)	-31.4 (-42.1 to -20.7)	-5.4 (-22.5 to 11.8)	0.54
Obesity class I, n=22 (BMI = 30.0-34.9)	-19.8 (-32.8 to -6.7)	-43.8 (-58.1 to -29.5)	-12.8 (-35.8 to 10.2)	0.28
Obesity class II & III, n=8 (BMI > 35)	-2.1 (-33.0 to 28.9)	-35.0 (-58.9 to -11.0)	-46.2 (-102.2 to 9.8)	0.22
Pre-obesity + obesity class I-III, n=75 (BMI > 25.0)	-20.9 (-29.5 to -12.3)	-31.7 (-41.4 to -22.0)	-8.1 (-23.1 to 6.9)	0.29
Obesity class I-III, n=30 (BMI >30.0)	-15.6 (-28.58 to -2.52)	-45.2 (-58.2 to -32.2)	-23.6 (-44.4 to -2.7)	0.034
**Change in total alcohol intake[b]**				
Normal weight, n=52 (BMI = 18.5 - 24.9)	-2514 (-2853 to -2174)	-946 (-1249 to -644)	463 (-159 to 1086)	0.15
Pre-obesity, n=45 (BMI = 25.0-29.9)	-1055 (-1336 to -774)	-1622 (-1966 to -1277)	-320 (-847 to 207)	0.23
Obesity class I, n=22 (BMI = 30.0-34.9)	-606 (-980 to -231)	-2327 (-2737 to -1917)	-238 (-963 to 486)	0.52
Obesity class II & III, n=8 (BMI > 35)	220 (-1386 to 1826)	-1427 (-2672 to -183)	-2122 (-4913 to 670)	0.25
Pre-obesity + obesity class I-III, n=75 (BMI > 25.0)	-727 (-1045 to -409)	-1918 (-2277 to -1559)	-701 (-1248 to -154)	0.013
Obesity class I-III, n=30 (BMI >30.0)	-378 (-963 to 207)	-2262 (-2847 to -1677)	-1205 (-2206 to -204)	0.026

Exploratory BM-subgroup analyses. Change in heavy drinking days, and total alcohol intake from baseline to week 26. Se Figure 4 & 5 in the main paper. Missing data were imputed with multiple imputations. Abbreviations: BMI, body mass index (calculated as weight in kilograms divided by height in square meters); [a]pp = percentage point.

Table 8: Posthoc analyses, clinical outcomes at the 6-month follow-up

From baseline/week 26 to the 6-month follow-up

Characteristic	Placebo group n = 65	Exenatide group n = 62	Estimated treatment difference, exenatide vs. placebo (95% Cl)	p-value
**Clinical, mean (95%Cl)**				
PEth from end of treatment (n=43)[a]	-0.15 (-0.40 to 0.11)	-0.14 (-0.38 to 0.10)	-0.05 (-0.47 to 0.37)	0.82
PACS from end of treatment (n=43)[a]	-2.6 (-4.5 to -0.7)	-1.8 (-3.6 to 0.0)	2.5 (-0.4 to 5.3)	0.09
AUDIT from end of treatment (n=43)[a]	-6.1 (-9.0 to -3.1)	-0.6 (-3.3 to 2.2)	5.1 (0.9 to 9.3)	0.019
Fagerströms from end of treatment (n=29)[b]	-0.10 (-1.12 to 0.93)	-0.27 (-1.19 to 0.66)	0.004 (-1.42 to 1.43)	1.00

[a]55 individuals participating in the 6-month follow-up, minus 12 individuals receiving various treatment for AUD. [b]all individuals who participated in the 6-month follow-up and had been smoking during the trial. No individuals were excluded due to other medication at the time of the assessment. PEth, Phosphatidylethanol; PACS, Penn Alcohol Craving Scale; AUDIT, Alcohol Use Disorders Identification Test and the Fagerström Test for Nicotine Dependence.

Table 9: Pre-specified sensitivity analysis

Change in endpoints from baseline to week 26

**Characteristic**	**Placebo group n = 65**	**Exenatide group n = 62**	**Estimated treatment difference, exenatide vs. placebo (95% Cl)**	**p-value**
**Clinical, mean (95 % Cl)**				
**Per protocol, n=58**				
Heavy drinking days (pp[a])	-29.9 (-40.2 to -19.5)	-20.0 (-31.5 to -8.5)	3.0 (-12.7 to 18.7)	0.70
Total alcohol consumption (g/30 days)	-1484 (-1879 to -1088)	-1275 (-1714 to -836)	-49.2 (-643 to 544)	0.87
Days without any alcohol consumption (pp[a])	26.4 (15.3 to 37.5)	19.2 (6.9 to 31.6)	-6.6[b] (-23.2 to 10.1)	0.43
**Imputation of full baseline value for all dropouts, n=127**				
Heavy drinking days (pp[a])[c]	-13.5 (-20.0 to -7.1)	-8.4 (-15.0 to -1.8)	4.8 (-4.5 to 14.0)	0.31
Heavy drinking days (pp[a])[d]	-15.7 (-22.8 to -8.6)	-13.6 (-20.8 to -6.3)	1.4 (-8.8 to 11.6)	0.78
Total alcohol consumption (g/30 days)[c]	-609 (908to -310)	-535 (-841 to -228)	58 (-370 to 486)	0.79
Total alcohol consumption (g/30 days)[d]	-647 (-964 to -331)	-738 (-1062 to -414)	-110 (-563 to 343)	0.63
Days without any alcohol consumption (pp[a])[c]	12.1 (6.0 to 18.1)	8.2 (2.0 to 14.4)	-4.3[b] (-13.0 to 4.4)	0.33
Days without any alcohol consumption (pp[a])[d]	13.3 (6.9 to 19.8)	7.6 (1.0 to 14.1)	-6.4 [b] (-15.7 to 2.8)	0.17
**Imputation of half baseline value for all dropouts, n=127**				
Heavy drinking days (pp[a])[c]	-29.3 (-34.1 to -24.4)	-26.4 (-31.4 to -21.4)	1.8 (-5.3 to 8.8)	0.61
Heavy drinking days (pp[a])[d]	-27.0 (-33.2 to -20.7)	-22.0 (-28.4 to -15.5)	4.0 (-5.0 to 13.0)	0.38
Total alcohol consumption (g/30 days)[c]	-1268 (-801to -416)	-1258 (-732 to -338)	-25.0 (-300 to 251)	0.86
Total alcohol consumption (g/30 days)[d]	-1159 (-1394 to -923)	-1142 (-1383 to -901)	-19 (-356 to 318)	0.91
Days without any alcohol consumption (pp[a])[c]	3.4 (-3.7 to 10.5)	0.1 (-7.2 to 7.3)	-4.6 [b] (-14.8 to 5.5)	0.36
Days without any alcohol consumption (pp[a])[d]	6.0 (-1.2 to 13.2)	2.3 (-5.1 to 9.7)	-5.1 [b] (-15.4 to 5.2)	0.33

[a]pp= percentage points, [b]a reduction indicates fewer 0-days, [c]imputation of full/half baseline value as endpoint, for all lost to follow-up and the 25 participants with a premature week 26, [d] imputation of full/half baseline value as endpoint, for all lost to follow-up, but not the 25 participants with a premature week 26.

Table 10: Inclusion and exclusion criteria

Inclusion Criteria	Exclusion criteria
Informed oral and written consent*	Severe psychiatric disorder, defined as a diagnosis of schizophrenia, paranoid psychosis, bipolar disorder, or mental retardation*
	A history of delirium tremens or alcohol withdrawal seizures*
Age 18 - 70 years (both included)*	No serious withdrawal symptoms at inclusion (a score higher than 9 on the Clinical Institute Withdrawal Assessment of Alcohol Scale, Revised (CIWA-Ar)) at baseline examinations
Diagnosed with Alcohol Use Disorder according to the criteria of International Classification of Diseases (ICD) 10, World Health Organization and DSM-5	Present or former neurological disease including traumatic brain injury*
Alcohol use disorder identification test (AUDIT) score >15	Type 1 diabetes, type 2 diabetes, or HbA1c ≥48 mmol/l at inclusion*
At least 5 days of heavy alcohol drinking, defined as having alcohol consumption over 60/48 (men/women) g of alcohol per day in the past 30 days before inclusion measured by the TLFB- method.	Females of childbearing potential who are pregnant, breastfeeding, or have the intention of becoming pregnant within the next nine months (26 weeks plus three months after discontinuation of Bydureon®), or are not using contraceptives (during the whole study period) considered as highly effective*
	Pregnancy (serum hCG > 3 at inclusion)*
	Impaired hepatic function (liver transaminases >3 times the normal upper limit)*
	Impaired renal function (eGFR < 50 ml/min and/or microalbuminuria)*
	Impaired pancreatic function (any history of acute or chronic pancreatitis and/or amylase > two times upper limit)*
	S-triglycerides > 10 mmol/l*
	Former medullary thyroid carcinoma (MTC) and/or family history with MTC and/or Multiple Endocrine Neoplasia syndrome type 2 (MEN 2)*
	Cardiac problems defined as decompensated heart failure (NYHA class III or IV), unstable angina pectoris, and/or myocardial infarction within the last 12 months*
	Uncontrolled hypertension (systolic blood pressure >180 mmHg, diastolic blood pressure >110 mmHg)*
	Concomitant pharmacotherapy against alcohol dependence including disulfiram, naltrexone, acamprosate, and nalmefene or treatment with any of these compounds within one month before inclusion*
	Concomitant pharmacotherapy with dopamine active drugs, such as some types of Attention Deficit Hyperactivity Disorder (ADHD) medication (methylphenidate)*
	Receiving any investigational drug within the last three months*
	Use of weight-lowering pharmacotherapy within the preceding three months*
	Any other active substance use defined as a DUDIT-score > 6 (for men) >2 (for women) *and* fulfilling the criteria for a dependence of the substance according to the criteria of International Classification of Diseases (ICD) 10 (except nicotine)*
	BMI <18,5 kg/m^2*
	Hypersensitivity to the active substance or any of the excipients*
	Unable to speak and/or understand Danish*
	Any condition that the investigator feels would interfere with trial participation*
*In- and exclusion criteria for healthy controls	For patients undergoing brain scans:<br>- Contraindications for undergoing an fMRI scan (magnetic implants, pacemaker, claustrophobia, etc.)*<br>Contraindications for undergoing a SPECT-scan (allergy towards iodine, radiation exposure, excluding background radiation but including diagnostic x-rays and other medical exposures, exceeding 10 mSv in the last 12 months

Table 11: Schedule of assessments

Assessors of all outcomes were medical doctors or medical students with a minimum of five years of training. All patients who discontinued prematurely and participated for at least eight weeks were encouraged to complete the week 26 follow-up visit. Only patients who completed the week 26 follow-up were invited to the six-month follow-up.

Assessments	Screening (week 0)	Follow-up (week 4)	Follow-up (week 12)	Follow-up (week 20)	Follow-up (week 26)	Follow-up (6 months)
**Diagnostic interview**	x					
**Adverse Reactions**		x	x	x	x	
**Cognition** SCIP-test*	x	x			x	
**Somatic examination***	x	x	x	x	x	
**Biobank** (se list below)	x				x	
**Safety laboratory tests*** (see list below)	x	x	x	x	x	x
**Quality of life questionnaires** (SF-36, SCL-92, MDI)	x				x	
**Psychosocial information**	x				x	
**AUDIT* questionnaire**	x				x	x
**DUDIT* questionnaire**	x				x	
**CIWA-Ar score**	x					
**TLFB-schedule**	x	x	x	x	x	x
**PACS questionnaire**	x		x		x	x
**Fagerströms questionnaire**	x	x	x	x	x	x
**Alco-Life questionnaire**						x
**fMRI + urine toxicology screening*** (subgroup)	x				x	
**SPECT** (sub group)	x				x	

Abbreviations: SCIP, Screen for Cognitive Impairment in Psychiatry test; SF-36, Short-Form Health Survey; SCL-92, Symptom Checklist; MDI: Major Depression Inventory Test; AUDIT, Alcohol Use Disorders Identification Test; DUDIT, Drug Use Disorders Identification Test; CIWA-AR: Clinical Institute Withdrawal Assessment of Alcohol Scale, Revised; TLFB: Time Line Follow Back schedule; PACS, Penn Alcohol Craving Scale; fMRI: functional magnetic resonance imaging; SPECT: single-photon emission computed tomography scan. *examinations of the healthy controls (n=25). Biobank: fibroblast growth factor 21 (FGF-21), plasma exenatide level- and antibodies, collagen type 1 C-telopeptide (CTX), procollagen type 1 N-terminal propeptide (P1NP), urinary biomarkers of oxidative stress, and blood- and urine sample for the biobank of future research. Laboratory test week 0 and 26: haemoglobin, mean corpuscular volume (MCV), leukocytes, leukocytes (differential count), thrombocytes, international normalized ratio (INR), glycated haemoglobin (HbA1c), amylase, albumin, gamma-glutamyl transferase (GGT), alanine aminotransferase (ALAT), cholesterol, triglycerides, high-density lipoprotein (HDL), low-density lipoprotein (LDL), vitamin D, cobalamin, sodium (Na+), potassium (K+), creatinine, thyroid-stimulating hormone (TSH), serum hCG, estimated glomerular filtration rate (eGFR), phosphatidylethanol (PEth), and urine albumin/creatinine ratio. Laboratory tests week 4 and 20: PEth, amylase. Laboratory tests week 12: phosphatidylethanol (PEth), amylase, ALAT, GGT, and eGFR. Laboratory test 6-month follow-up: PEth

Figure 2: Study flow diagram

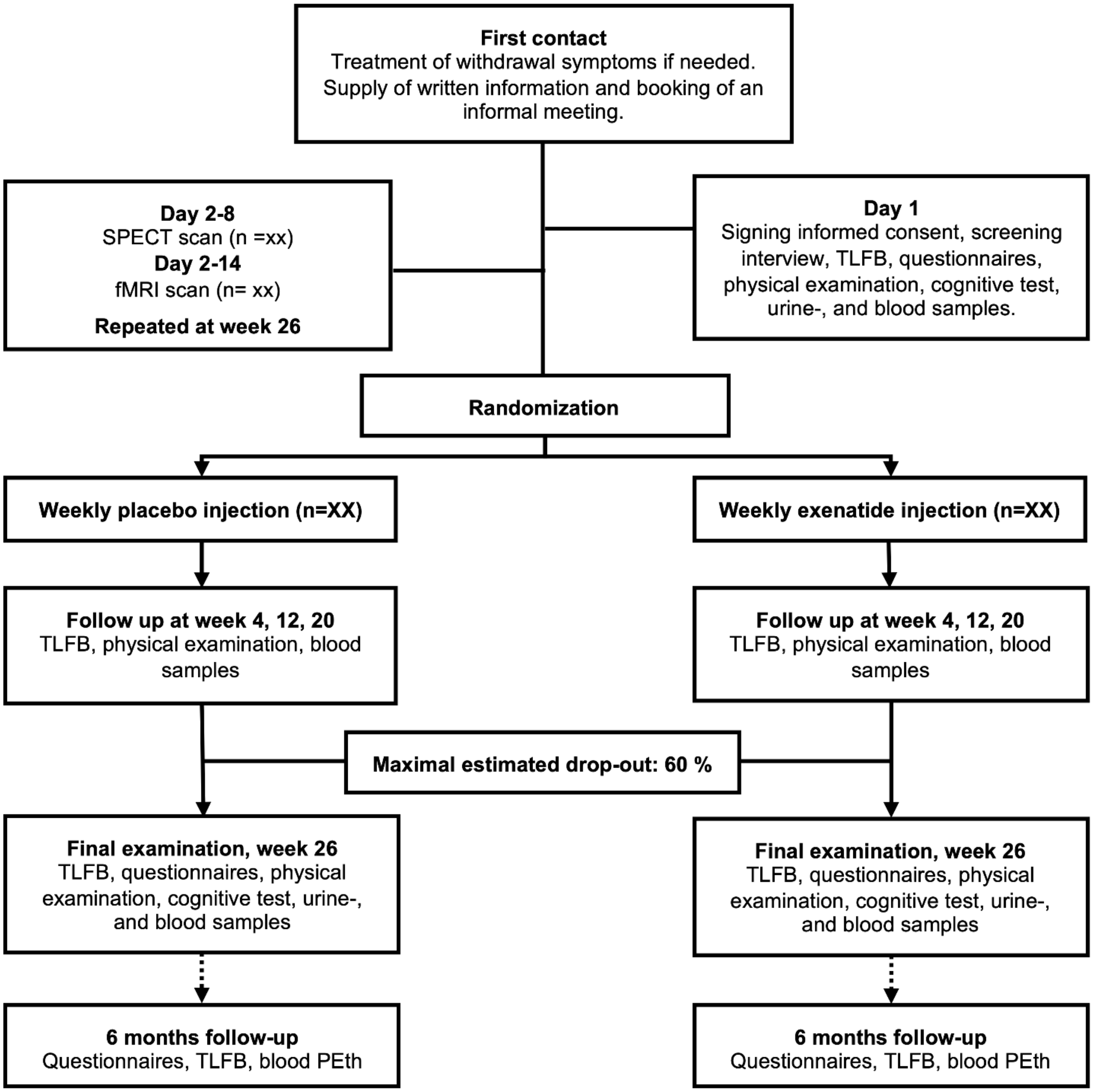

Study flow diagram. Abbreviations: SPECT, Single-photon emission computed tomography; fMRI, functional Magnetic Resonance Imaging; PEth, Phosphatidylethanol; TLFB, Time-Line Follow-Back Schedule

Table 12: Summary of protocol amendments

Protocol version	Amendment	Approval from the Danish Health Authority	Approval from the Ethics Committee
**Version 2.0**	First final protocol	March 20, 2017	Not approved
**Version 3.0**	First final protocol revised	Only Ethics Committee had to approve the changes.	April 12, 2017
**Version 4.0**	Modification of inclusion criteria:<br>Age 18-64 → 18-70 years<br><br>Acceptance of CBT for the last three months.<br><br>Acceptance of concomitant therapy for the last month instead of three months before inclusion.<br><br>Other:<br>Removal of plasma lipase and ASAT<br><br>+ The Fagerström Test for Nicotine Dependence at week 0, 4, 12, 20, 26, 6-months.	September 22, 2017	October 10, 2017
**Version 5.0**	fMRI-scan: +urine screen for drugs, but the removal of per mile restrictions.<br><br>Exclusion criteria:<br>Diastolic blood-pressure 100 mmHg → 110 mmHg<br>Removal of therapy with anticoagulants<br><br>Other:<br>Plasma bone markers.	February 21, 2018	January 26, 2018
**Version 6.0**	Prolongation of the study period<br><br>+ 6-month follow-up for participants who have participated for more than eight weeks	July 2, 2018	July 4, 2018
**Version 7.0**	Inclusion of 30 patients more.<br><br>Prolongation of the study period.	June 28, 2019	July 4, 2019
**Version 8.0**	Plasma FGF-21 and exenatide concentrations	April 23, 2020	May 29, 2020

Abbreviations: CBT, Cognitive behavioral therapy; ASAT, Aspartate aminotransferase; fMRI, functional magnetic resonance imaging; FGF-21, Fibroblast growth factor 21.

Figure 3: Flowchart – initial meeting

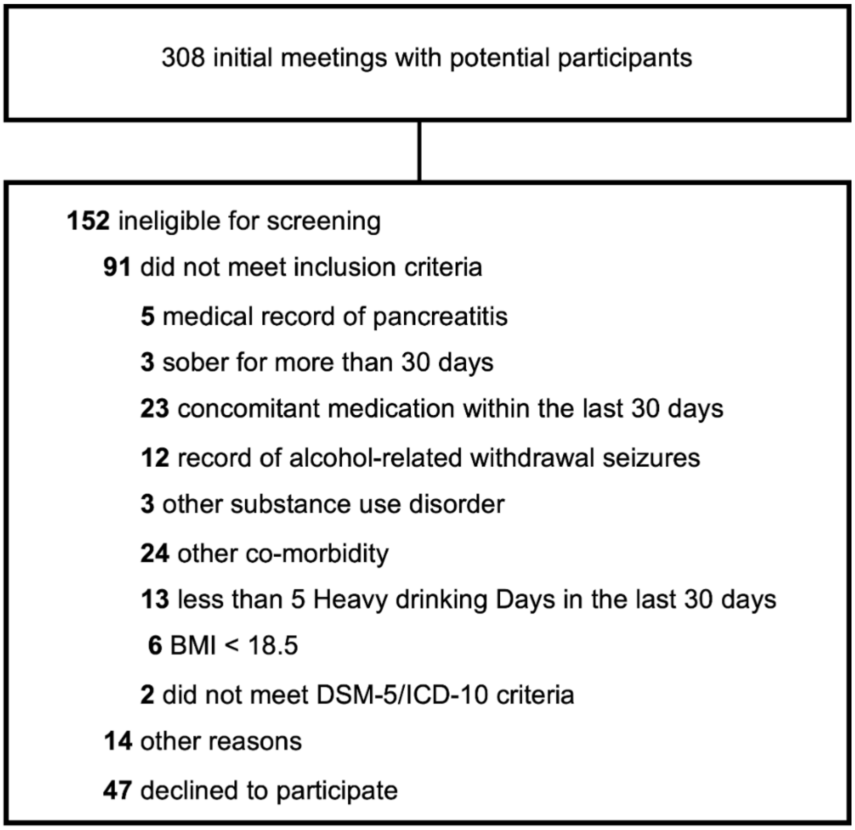

Appendix 1: fMRI Alcohol cue-reactivity & spatial working memory

fMRI Method

All participants in the main trial were invited to have an fMRI scan performed before randomization. Patients who underwent supervised detoxification with the long-lasting benzodiazepine chlordiazepoxide had to have finished the treatment for 12 days, i.e.,> 5 x elimination half-life (t½) before the scan session (4).

The 25 healthy controls matched with gender, age, and education level, had a somatic examination and the same blood tests, urine pregnancy test, and a rapid response urine test for drugs as the patients. An AUDIT score >8 resulted in exclusion. The healthy controls were only scanned once and had a Screen for Cognitive Impairment (SCIP3) test before receiving instructions for the scanner. For baseline characteristics, see appendix 1: Table 17 and Figure 7 for a flowchart of patients included in the fMRI subgroup.

Assessment of the fMRI scan

Before receiving standardized instructions outside the scanner, a breath alcohol test (Lion alcolmeter SD-400) and neurological test were performed. As a safety precaution, a permille between 0‰ and 1.0‰ led to a clinical assessment to decide whether the patient could be scanned. The patients provided a urine sample (rapid response multi-drug test panel from BTNX Inc) for cocaine (300), amphetamine (1000), Tetrahydrocannabinol (50), methadone (300), opioids (2000), benzodiazepine (300) (cut-off ng/mL) before entering the scanner. A positive urine sample (dummy coded “no” = 0, “yes” = 1) was registered as a potential covariate. No corrections were made in the final analysis due to only negative tests, except for traces of benzodiazepine.

To investigate if the patients ingested alcohol in the bathroom just before entering the scanner, all patients had to do a new breath alcohol test after the fMRI scanning session ended. No patients had rising values during the time in the scanner.

fMRI acquisition

The MRI scan was performed using a 3 Tesla Siemens Prisma scanner with a 64-channel head coil at the Copenhagen University Hospital, Rigshospitalet. The scanning sequence included a localizer, a T1-weighted structural image, gradient field mapping, resting state, and two fMRI tasks: alcohol cue-reactivity (ALCUE) and spatial working memory (N-back task). The total duration of time in the scanner for every individual was approximately 45 minutes. For the fMRI tasks, images were presented on an opaque screen, which the participants viewed through an angled mirror. Task presentation was performed using E-prime (version 2.0, Psychology Software Tools, Inc., Sharpsburg, PA). Blood oxygen level-dependent (BOLD) fMRI was acquired using a T2*-weighted gradient echo spiral echo-planar (EPI) sequence with an echo time (TE) of 30 ms, repetition time (TR) of 2 s, and flip angle of 90°. The fMRI volumes consisted of 32 slices with a slice thickness of 3 mm and 25% gaps in-between, field of view (FOV) of 230×230 mm using a 64×64 grid. A total of 357 volumes were acquired for the ALCUE task and 230 for the N-back task. The T1-weighted structural images (TR=1900 ms; TE=2.58 ms; flip angle=9°; distance factor=50%; FOV=230 × 230 mm; slice thickness=0.9 mm) were used to improve the registration of the BOLD images to a standard MNI template. A standard B0 field map sequence was acquired with the same FOV and resolution as the fMRI sequences (TR=400 ms; TE1 = 4.92; TE2=7.38 ms; flip angle=60°) and used for geometric distortions correction of the BOLD images. The quality of the MRI scans was ascertained by visual inspection of all individual images. To minimize head motion, participant's heads were fixated with foam wedges

fMRI Alcohol cue reactivity (ALCUE)

Hypotheses - ALCUE

A. Exenatide will modulate blood oxygenation level-dependent (BOLD) signal changes during a cue-reactivity task in reward processing regions in the ventral striatum (nucleus accumbens), the putamen, and the caudate
B. We hypothesized a larger decrease in subjective cue-induced alcohol craving for the patients in the exenatide group than in the placebo group

Task description – ALCUE

Sixty alcohol-related and 45 neutral stimuli were presented in a pseudo-randomized block design. Each block consisted of five stimuli, which were presented for four seconds each (5). Alcohol-related pictures were taken from a validated picture series (6), and neutral cues were taken from the International Affective Picture System (IAPS) (7). After every block (both alcohol- and neutral-pictures), participants were asked to evaluate their craving on a visual analog scale from zero (no craving at all) to 100 (severe craving) (5). Participants had a maximum of 10 seconds to perform the evaluation, after which a black fixation cross was presented on a white background for a variable period such that the total time including the evaluation and post-evaluation fixation, was 15 seconds. The entire task duration was 12 minutes (6) (Figure 4).

Figure 4

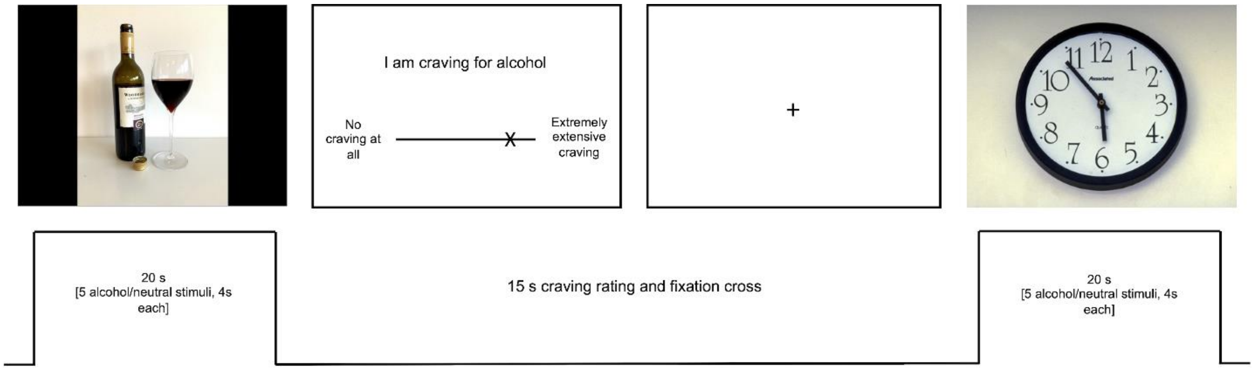

fMRI preprocessing – ALCUE

Preprocessing and statistical analysis of ALCUE data was performed using SPM 12 (Wellcome Department of Cognitive Neurology, London, United Kingdom). The first five scans were excluded to prevent saturation effects. The remaining 357 scans underwent image realignment and unwarping, co-registration with the T1 structural image, spatial normalization to an MNI template, and spatial smoothing (Gaussian kernel, 8 mm full-width-half-maximum). One participant was excluded due to head motion during the scan session in excess of 3 mm.

Statistical analysis – ALCUE

Statistical analyses of the preprocessed fMRI data on the first (individual) level were performed by modeling the different conditions (task-related boxcar functions convolved with the

hemodynamic response function) as explanatory variables within the context of the general linear model (GLM) on a voxel-by-voxel basis with SPM12. Data were high-pass filtered at, 128 seconds (i.e., 0.008 Hz). We calculated an image for the contrast 'alcohol versus neutral blocks' for each participant. Individual contrast images were used for the second-level analysis to identify brain regions with differential activation to alcohol cues across groups. The second-level analysis was performed using a full factorial model to test the interaction between time and group (T1, T2, placebo, exenatide), an ANOVA to compare all three groups, including healthy controls (placebo, exenatide, HC), and a two-sample t-test for the post-hoc analyses to compare groups (placebo, exenatide) and within a group across time (placebo/exenatide: T1, T2).

To control for multiple statistical testing in the whole-brain analyses, the probability for a family-wise error (FWE) was set to 0.05. Using 25000 Monte Carlo Simulations in AFNI's 3dClustSim (Analysis of Functional NeuroImages, www.afni.nimh.nih.gov/) a voxel-wise-threshold of $P < 0.001$ in combination with a cluster-extend-threshold of $k \geq 101$ for the two-sample t-tests, $k \geq 109$ for the ANOVA, and $k \geq 104$ for the full factorial model with individuals completing per-protocol, and $k \geq 105$ for the full factorial model including individuals per-protocol plus individuals with a premature rescan, were determined with an estimation of smoothness implemented in the SPM software. The cluster images were constructed with the MRIcroGL software.

Further regions of interest (ROI) analyses were conducted within the putamen, caudate, nucleus accumbens (NAc) dorsal- and ventral striatum. All regions were determined based on previous studies (5,8). The nucleus accumbens-, dorsal- and ventral striatum-, putamen-, and caudate-masks were acquired from the WFU PickAtlas. For ROI data-aggregation, a self-written SPM toolbox (by S.V.) was used, which was described previously by Reinhard et al. (9), and here, the measure „sum_indiv_t_norm" was chosen, corresponding to the sum of t-values exceeding an individual threshold, "defined as 50% of the of the mean of the 5% highest t-values in a given individual's SPM-t map to unbias subjects with high overall t-values" (9). Due to our detailed a priori specified hypotheses, no adjustments for multiple testing were conducted for the ROI aggregation data analyses.

Craving and ROI aggregated data were analyzed using SPSS (Statistical Package of the Social Sciences, IBM SPSS Statistics for Windows, Version 25.0. Armonk, NY: IBM Corp). Similar to the whole-brain analysis, the aggregated ROI data were analyzed using a repeated-measures ANOVA, including factors group and time and an independent sample T-test comparing groups

(placebo and exenatide). Subjective craving data were analyzed with a one-way ANOVA with factor group (placebo, exenatide, HC).

<u>Table 13</u>: Reduced cue-induced activation in the exenatide group (n=10) compared to the placebo group (n=12) at the week 26 re-scan (contrast alcohol - neutral stimuli, combined voxel-wise- [P < 0.001] and cluster-extent threshold [k >= 101 voxels], corresponding to pFWE < 0.05) (Figure 7 in the main paper)

**Cluster**	**Side**	**Lobe**	**Region (aal)**	**Number of voxels in region**	**Cluster size**	**x**	**y**	**z**	**tmax**
1			Caudate	38	111	0	0	4	5,3612
2	R	Frontal	Middle Frontal Gyrus	123	124	36	20	48	4,6472

Table 14: Decrease of cue-induced activation over time in the exenatide group (n=10) from baseline to the week 26 re-scan, (contrast alcohol - neutral stimuli, combined voxel-wise- [P < 0.001] and cluster-extent threshold [k >= 104 voxels], corresponding to pFWE < 0.05) (Figure 5)

Cluster	Side	Lobe	Region (aal)	Number of voxels in region	Cluster size	x	y	z	tmax
1	L	Temporal	Middle temporal gyrus	125	158	-56	-10	-12	4,496
			Superior temporal gyrus	29					
2	R	Temporal	Middle temporal gyrus	90	136	60	6	-22	4,590
			Temporal pole: middle temporal gyrus	24					
			Superior temporal gyrus	22					
3	R	Limbic	Hippocampus	97	218	28	-20	-20	5,533
			Parahippocampal gyrus	88					
			Amygdala	9					
			Fusiform gyrus	4					
4	L		Parahippocampal gyrus	81	553	-6	-12	-20	5,1571
			Hippocampus	74					
			Amygdala	21					
			Middle temporal gyrus	15					
			Temporal pole: superior temporal gyrus	13					
			Cerebellum	2					
			Fusiform gyrus	1					
			Cerebellum	1					

Figure 5

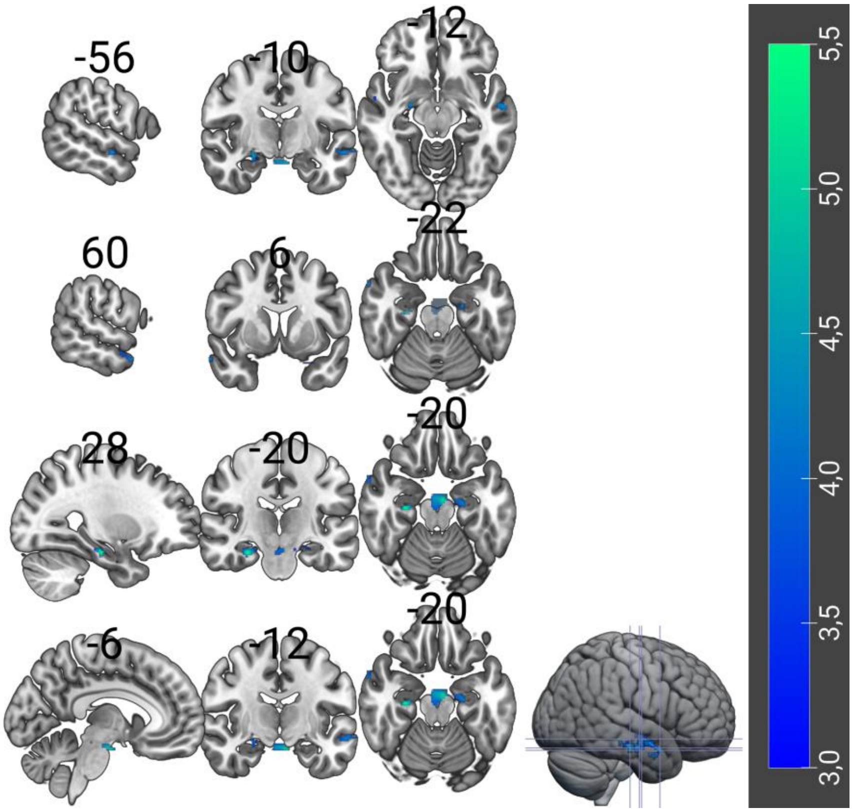

Table 15: Decrease of cue-induced activation over time in the exenatide group (n=17) from baseline to the week 26 re-scan including premature re-scans (contrast alcohol - neutral stimuli, combined voxel-wise- [P < 0.001] and cluster-extent threshold [k >= 105 voxels], corresponding to pFWE < 0.05) (Figure 6)

cluster	Side	Lobe	Region (aal)	Number of voxels in region	Clus-ter size	x	y	z	tmax
1	R	Limbic	Hippocampus<br>Parahippocampal gyrus	88<br>59	156	24	-12	-18	4,949
**2**	**L**		**Hippocampus**<br>**Parahippocampal gyrus**	**56**<br>**15**	**346**	**0**	**-14**	**-20**	**4,527**

Figure 6

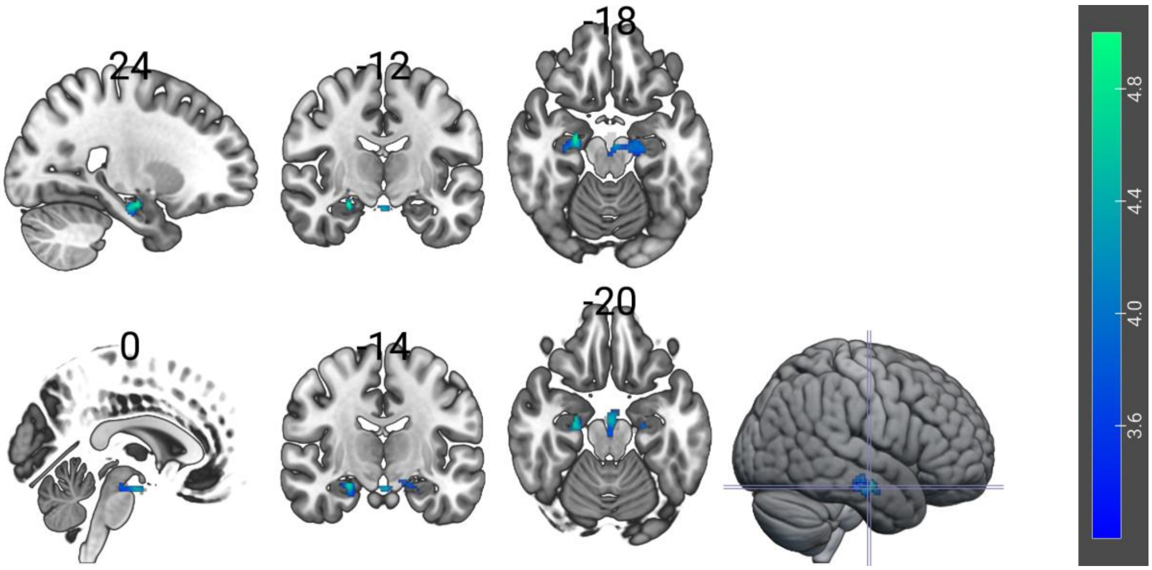

fMRI spatial working memory (N-back Task)

Hypotheses – N-back task:

A. exenatide will increase working memory-related activity in the dorsal PFC
B. the increased dorsal PFC activity will correlate with improvement in cognitive performance

Task description - N-back task:

The participants viewed a black screen where a yellow circle would appear randomly in a 5x5 grid for 300 ms, followed by an empty grid for 1200 ms. During the 0-back condition, the participants had to press a button on a response pad whenever the ball appeared in any grid corners. In the 1-back and 2-back conditions, the participants had to indicate whenever the ball appeared in the same grid square as one trial or two trials back, respectively. Each of the three condition blocks had 14 trials (three targets), with blocks presented five times in a pseudo-random order. Each block was interleaved by a fixation cross (8 seconds). The total task duration was 7 minutes and 35 seconds.

Regions of interest – N-back task

Based on our a priori hypothesis, we constructed a dPFC mask using FSLView 4.0.1 on a standard MNI template based on the Harvard-Oxford cortical structural Atlas probabilistic maps (10) by including bilateral superior and medial frontal gyri and the superior portions of the anterior division of the cingulate gyrus and the frontal poles thresholded at 5%. The ventral border of the dPFC was defined by the plane separating the dorsal from the ventral regions of medial PFC (MNI z>5), defined according to Veit et al., 2010 (11). To investigate the neural mechanisms of treatment-related improvement in executive function, we defined a spherical (10 mm radius) ROI for the right dorsolateral prefrontal cortex (dlPFC) centered on the peak dlPFC region (MNI coordinates: x=40, y=34, z=29) involved in active WM processes as reported in a meta-analysis (12).

fMRI preprocessing - N-back task:

Functional MRI data processing was performed with the FMRI Expert Analysis Tool (FEAT; version 6.01) part of FMRIB's Software Library (FSL; www.fmrib.ox.ac.uk/fsl). Pre-processing involved image B_0 field distortion correction with acquired field map image, realignment of the

acquired fMRI volumes to the first one in the series, non-brain removal, spatial normalization to a Montreal Neurologic Institute (MNI) template, and spatially smoothing (Gaussian kernel, 5 mm full-width-half-maximum). The time series in each session were high pass-filtered (min 0.01).

Statistical analysis - N-back task:

At subject level, the n-back task was modeled using a block design with three conditions: 2-back, 1-back, and 0-back. The boxcar functions of the three events were convolved with a double-gamma hemodynamic response function, and we added temporal derivatives for improved fit to the data. Two contrasts of interest were defined: 2-back>1-back (high load specific WM-related activity) and 2-back>0-back (general WM-related activity).

For the group analysis, we first estimated task activations at baseline for the healthy control group by including the two contrasts of interest in one-sample t-tests. Secondly, we estimated group-by-time interaction effects for the contrasts of interest using a two-way mixed effect repeated measures ANOVA model (group factor: treatment group, time factor: baseline and follow-up). The significance level for clusters was set at $p<.05$ corrected for multiple comparisons using Gaussian Random Field (GRF) theory subsequent a cluster-forming threshold of $z=2.57$ ($p<.005$). The models were estimated twice, first restricting the search volume to the dPFC mask and secondly at whole-brain level.

For the ROI analysis, we extracted the mean percent BOLD signal change from a right dlPFC ROI, in all participants. The BOLD signal used estimated the longitudinal effect of exenatide vs. placebo in a repeated measures general linear model implemented in SPSS v25 (IBM, Armonk, New York, United States). We further performed a correlation analysis to explore possible associations between the baseline to follow-up change in total SCIP test performance and change in dlPFC response.

Results - N-back task

The healthy control group activated a wide-spread bilateral fronto-parietal network to 2-back>0-back including the bilateral dlPFC, and a left parietal cortex region to 2-back>1-back (Figure 4C, Table S15). The longitudinal analyses of the patients revealed two clusters within the hypothesized dPFC mask showing a significant treatment group-by-time interaction effect. Whole-brain analyses revealed no additional clusters with a significant treatment group-by-time interaction

effect. The task-related working memory activations in the right dlPFC ROI analysis did not show differential longitudinal change between groups ($p=0.122$), and the longitudinal change in task-related activations in the exenatide group did not correlate with the change in total SCIP score ($p=0.247$).

Table 16:

Region	Hemi-sphere	BA	Cluster size	x	*y*	z	Z-stat	Cluster p
**Healthy Controls (baseline)**								
**2-back > 0-back**			63910					<0.001
Supramarginal Gyrus	R	40		42	-38	40	7.53	
Middle Frontal Gyrus	R	6		34	6	54	7.01	
Middle Frontal Gyrus	R	8		34	12	58	7.00	
Lateral Occipital Cortex	R	7		34	-60	40	6.97	
Lateral Occipital Cortex	R	18		32	-86	8	6.96	
**2-back > 1-back**								
Supramarginal Gyrus	R	40	4056	40	-38	46	5.85	<0.001
Lateral Occipital Cortex	L	19	3171	-32	-86	20	4.63	<0.001
Middle Frontal Gyrus	L	6	793	-28	0	54	3.95	<0.001
Orbitofrontal Cortex	R	47	342	32	26	-8	4.64	0.048
**Treatment group (group × time interaction)**								
Frontal pole	R	46	290	34	54	20	4.17	0.002
Superior Frontal Gyrus	R	32	334	4	46	46	3.73	0.001

BA=Broadman Area, Cluster size=number of voxels (2×2×2 mm) in the significant cluster, x,y,z = MNI coordinates of local maxima, Z-stat=max statistical Z values for voxel, p = corrected p value of the cluster.

Figure 7. fMRI flowchart

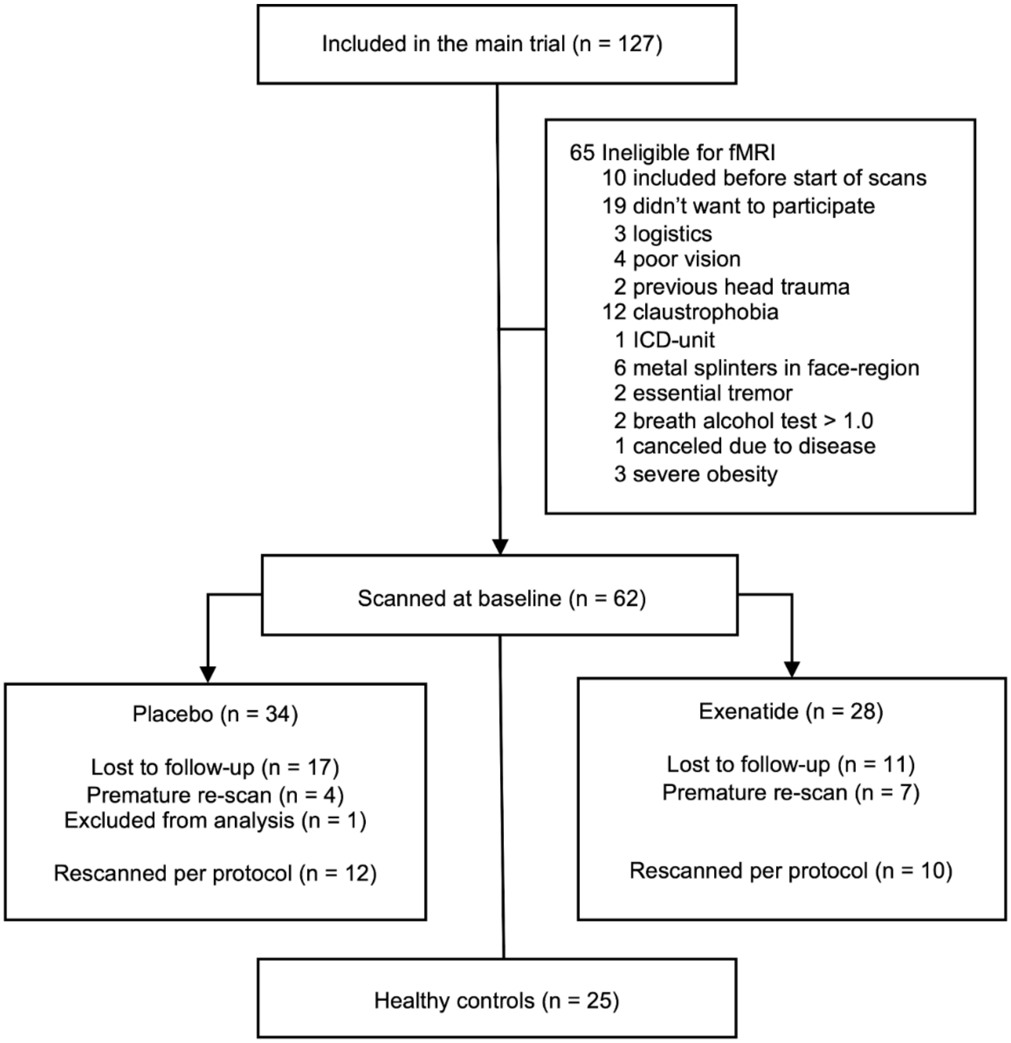

Table 17. fMRI Baseline characteristics

	**placebo** n=33	**exenatide** n=28	**Overall** n=61	**Healthy controls** n=25
**Age** mean (SD)	49.2(11.0)	51.4 (10.8)	50.2 (11.0)	49.3(9.01)
**Men**	20 (60.6 %)	16 (57.1 %)	36(59.0 %)	15(60 %)
**Lefthanded**	6(18.2 %)	1(3.6 %)	7(11.7 %)	4(16 %)
**Education (years)** mean (SD)	14.7(2.50)	13.5 (2.43)	14.1 (2.51)	14.6(2.14)
**DSM-5** mean (SD)	8.12(2.22)	6.96(2.30)	7.59(2.30)	-
**Heavy drinking days**[a] mean (SD)	18.1 (8.80)	16.9 (9.08)	17.6(8.88)	-
**Units of alcohol**[a+b] mean (SD)	199(129)	174(95.0)	188(115)	-
**Days without alcohol intake**[a] mean (SD)	9.39(8.20)	8.79 (7.93)	9.11(8.02)	-
**AUDIT-score** Mean (SD)	26.7(4.44)	24.9 (4.92)	25.9(4.72)	3.32 (1.80)

[a]in the last 30 days, [b]one unit = 12 grams of pure alcohol; DSM-5: Diagnostic and Statistical Manual of Mental Disorders 5; AUDIT: alcohol use disorder identification test.

Appendix 2: single-photon emission computed tomography – SPECT scan

SPECT Method

All patients included in the main trial were invited to have a SPECT scan performed. Due to an expected upregulation of DAT under abstinence (13), the SPECT scan was completed within seven days of inclusion. The final sample size was 46 baseline scans, 16 rescans per protocol in total, and five premature re-scans (flowchart, Appendix 2: Figure 8). One baseline scan was excluded due to technical issues. All patients included in the SPECT sub-study were also included in the fMRI-sub-study, which involved an anatomical brain MRI. No patients had any brain abnormalities leading to exclusion.

A breath alcohol test and rating on the CIWA-Ar scale for alcohol withdrawal symptoms were performed at arrival. If the CIWA-Ar score were above 9, the scan was rescheduled. The breath-alcohol test was repeated just before entering the scanner, and the result was registered as a potential covariate. At the baseline scan, two patients had an alcohol permille below 0.33 just before entering the scanner, and at the rescan, one patient had an alcohol permille of 0.63. For baseline characteristics, see Appendix 2: Table 19.

SPECT tracer injection:

The DAT ligand [123I]-FP-CIT was administrated as a bolus injection. All patients received 200 mg potassium perchloride intravenously five min before the [123I]-FP-CIT injection to block thyroidal uptake of free radioiodine. The bolus size was approximately 185 MBq, based on a previous study (14).

SPECT equipment:

SPECT imaging was performed with a triple-head IRIX camera (Philips Medical, Cleveland, USA) fitted with low-energy, general-all-purpose, parallel-holed collimators (spatial resolution 8.5 mm at 10 cm). The mean radius of rotation was 14-15 cm. The total time in the scanner was 60 min. The six SPECT acquisitions, each lasting 10 min, were obtained between 180 and 240 min after the 123I-FP-CIT injection. Reconstruction of the images was performed with a MATLAB 6.5 (MathWorks) based program in 128×128 matrices (2.33 mm pixels and identical slice

thickness) using standard filtered back projection with a low pass fourth-order Butterworth filter at 0.3 Nyquist (=0.64 c−1). The imaging energy window is positioned at 143-175 keV. High-energy photons of 123-I penetrated through the lead of the collimator, and Compton scatter in the scintillation crystal caused erroneous counts in the imaging energy window. A second energy window positioned at 184-216 keV was used to correct down-scattered photons in the imaging window. Before reconstruction, the projection images of the second energy window were subtracted from the imaging energy window with a weight of 1.1.

DAT quantification:

The binding potential (BPnd) of [123I]-FP-CIT was used as a measure of DAT availability. [123I]-FP-CIT BPnd was calculated as the ratio at steady-state of the concentration of specifically bound [123I]-FP-CIT (concentration of total [123I]-FP-CIT in a volume of interest (VOI) minus concentration of [123I]-FP-CIT in a reference region) to the reference region concentration of [123I]-FP-CIT. The cerebellum was used as the reference tissue devoid of DAT. For all patients, regional BPnd values were calculated in striatum, caudate, and putamen using an in-house developed algorithm DATquan (15).

SPECT Statistical analysis

The primary aim (1) in this sub-study was to assess the between-group difference of DAT availability after 26 weeks of treatment with either exenatide or placebo. To this end, we used a one-way analysis of covariance (ANCOVA), adjusting baseline DAT availability. The secondary aim (2) was to assess whether DAT availability differed in patients with AUD compared to healthy controls using ANCOVA adjusting for age.

SPECT results

All patients (N=45) had BPnd values within the normal range of our laboratory (16), and none had left/right hemisphere asymmetry nor clinical signs of neurological disease as assessed by a brief clinical neurological examination. See appendix 2, table 19, for baseline characteristics of the patients. Twenty-one healthy controls included in two previous studies (14,17) using the same scanner, tracer (FP-CIT), and quantification method (DATquan) were included in the analysis to compare baseline DAT availability in AUD with healthy controls.

Table 18

Adjusted and unadjusted means and variability for BPnd in striatum, caudate and putamen with baseline as a covariate.

		Adjusted		Unadjusted	
	N	Mean	SE	Mean	SE
**Striatum**					
placebo	9	4.368a	0.144	4.367	0.219
exenatide	7	3.882a	0.163	3.883	0.371
**Caudate**					
placebo	9	5.146b	0.164	5.165	0.272
exenatide	7	4.441b	0.186	4.416	0.344
**Putamen**					
placebo	9	4.748c	0.166	4.698	0.234
exenatide	7	4.104c	0.188	4.167	0.463

Covariates appearing in the model are evaluated at the following values: baseline striatum = 4.102a, baseline caudate = 4.739b, baseline putamen = 4.386c

__Figure 8, SPECT flowchart__

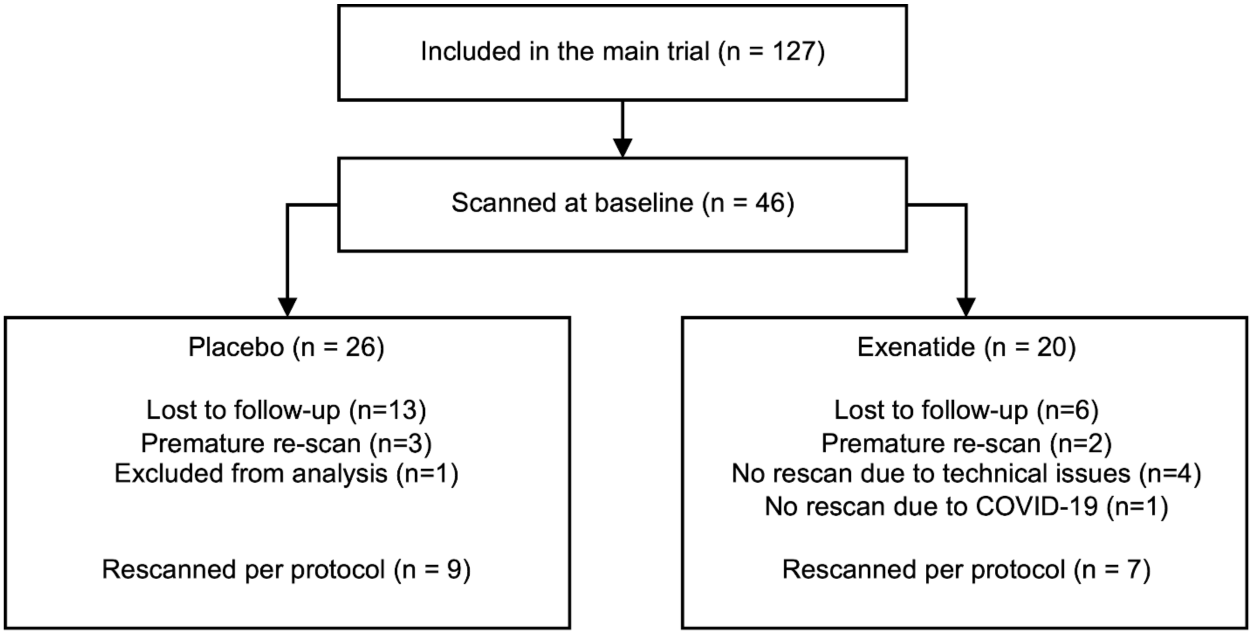

All patients included in the SPECT sub-study were also included in the fMRI sub-study. See the fMRI flowchart (Figure 7) for details of ineligibility.

Table 19, SPECT Baseline characteristics

	**Placebo group** n=25	**Exenatide group** n=20	**Overall** n=45
**Age** mean (SD)	54.5 (7.20)	52.0 (11.5)	53.4 (9.33)
**Men**	18 (72.0%)	11 (55.0%)	29 (64.4%)
**DSM-5** mean (SD)	8.24 (2.42)	6.70 (2.23)	7.56 (2.44)
**Heavy drinking days**[a] mean (SD)	18.7 (9.27)	16.0 (8.80)	17.5 (9.06)
**Units of alcohol**[a+b] mean (SD)	202 (138)	168 (91.8)	187 (120)
**Days without alcohol intake**[a] mean (SD)	8.40 (8.20)	8.10 (6.87)	8.27 (7.55)
**AUDIT-score** mean (SD)	26.1 (4.84)	25.0 (4.95)	25.6 (4.86)

[a]in the last 30 days, [b]one unit = 12 gram of pure alcohol; AUDIT: Alcohol Use Disorder Identification Test; DSM-5: Diagnostic and Statistical Manual of Mental Disorders 5.

Reference list

1. Søberg S, et al. FGF21, a liver hormone that inhibits alcohol intake in mice, increases in human circulation after acute alcohol ingestion and sustained binge drinking at Oktoberfest. *Mol Metab*. 2018;11(March):96-103. doi:10.1016/j.molmet.2018.03.010
2. Rasmussen ST, et al. Simvastatin and oxidative stress in humans: A randomized, Double-blinded, Placebo-controlled clinical trial. *Redox Biol*. 2016;9:32-38. doi:10.1016/j.redox.2016.05.007
3. RAND 36-Item Health Survey 1.0. 36-Item Short Form Survey (SF-36) Scoring Instructions. https://www.rand.org/health-care/surveys_tools/mos/36-item-short-form/scoring.html
4. Medscape. chlordiazepoxide. Accessed January 21, 2021. https://reference.medscape.com/drug/librium-chlordiazepoxide-342899#10
5. Vollstädt-Klein S, et al. Effects of cue-exposure treatment on neural cue reactivity in alcohol dependence: A randomized trial. *Biol Psychiatry*. 2011;69(11):1060-1066. doi:10.1016/j.biopsych.2010.12.016
6. Vollstädt-Klein S, et al. Initial, habitual and compulsive alcohol use is characterized by a shift of cue processing from ventral to dorsal striatum. *Addiction*. 2010;105(10):1741-1749. doi:10.1111/j.1360-0443.2010.03022.x
7. Lang, P.J., et al. International affective picture system (IAPS): Affective ratings of pictures and instruction manual. Technical Report A-8. *Tech Rep A-8*. Published online 2008:Tech. Rep. A-8. doi:10.1016/j.epsr.2006.03.016
8. Kiefer F, et al. Effects of d-cycloserine on extinction of mesolimbic cue reactivity in alcoholism: A randomized placebo-controlled trial. *Psychopharmacology (Berl)*. 2015;232(13):2353-2362. doi:10.1007/s00213-015-3882-5
9. Reinhard I, et al. A comparison of region-of-interest measures for extracting whole brain data using survival analysis in alcoholism as an example. *J Neurosci Methods*. 2015;242:58-64. doi:10.1016/j.jneumeth.2015.01.001
10. Desikan RS, et al. An automated labeling system for subdividing the human cerebral cortex on MRI scans into gyral based regions of interest. *Neuroimage*. 2006;31(3):968-980. doi:10.1016/j.neuroimage.2006.01.021

11. Veit R, et al. Aberrant social and cerebral responding in a competitive reaction time paradigm in criminal psychopaths. *Neuroimage*. 2010;49(4):3365-3372.
12. Wager TD, Smith EE. Neuroimaging studies of working memory: A meta-analysis. *Cogn Affect Behav Neurosci*. 2003;3(4):255-274.
13. Hansson AC, et al. Dopamine and opioid systems adaptation in alcoholism revisited: Convergent evidence from positron emission tomography and postmortem studies. *Neurosci Biobehav Rev*. 2019;106:141-164. doi:10.1016/j.neubiorev.2018.09.010
14. Ziebell M, et al. Serotonin Transporters in Dopamine Transporter Imaging: A Head-to-Head Comparison of Dopamine Transporter SPECT Radioligands 123I-FP-CIT and 123I-PE2I. *J Nucl Med*. 2010;51(12):1885-1891. doi:10.2967/jnumed.110.078337
15. Jensen PS, et al. Validation of a method for accurate and highly reproducible quantification of brain dopamine transporter SPECT studies. *J Nucl Med Technol*. 2011;39(4):271-278. doi:10.2967/jnmt.111.090324
16. Varrone A, et al. European multicentre database of healthy controls for [123I]FP- CIT SPECT (ENC-DAT): Age-related effects, gender differences and evaluation of different methods of analysis. *Eur J Nucl Med Mol Imaging*. 2013;40(2):213-227. doi:10.1007/s00259-012-2276-8
17. Thomsen G, et al. No correlation between body mass index and striatal dopamine transporter availability in healthy volunteers using SPECT and [123I]PE2I. *Obesity*. 2013;21(9):1803-1806. doi:10.1002/oby.20225
